# Associations between Single Nucleotide Polymorphisms in Cellular Viral Receptors and Attachment Factor-Related Genes and Humoral Immunity to Rubella Vaccination

**DOI:** 10.1371/journal.pone.0099997

**Published:** 2014-06-19

**Authors:** Iana H. Haralambieva, Nathaniel D. Lambert, Inna G. Ovsyannikova, Richard B. Kennedy, Beth R. Larrabee, V. Shane Pankratz, Gregory A. Poland

**Affiliations:** 1 Mayo Vaccine Research Group, Mayo Clinic, Rochester, Minnesota, United States of America; 2 Division of Biostatistics, Mayo Clinic, Rochester, Minnesota, United States of America; 3 Department of Pediatric and Adolescent Medicine, Mayo Clinic, Rochester, Minnesota, United States of America; 4 Program in Translational Immunovirology and Biodefense, Mayo Clinic, Rochester, Minnesota, United States of America; Wageningen University and Research Centre, Netherlands

## Abstract

**Background:**

Viral attachment and cell entry host factors are important for viral replication, pathogenesis, and the generation and sustenance of immune responses after infection and/or vaccination, and are plausible genetic regulators of vaccine-induced immunity.

**Methods:**

Using a tag-SNP approach in candidate gene study, we assessed the role of selected cell surface receptor genes, attachment factor-related genes, along with other immune genes in the genetic control of immune response variations after live rubella vaccination in two independent study cohorts.

**Results:**

Our analysis revealed evidence for multiple associations between genetic variants in the *PVR, PVRL2, CD209/DC-SIGN, RARB, MOG, IL6* and other immune function-related genes and rubella-specific neutralizing antibodies after vaccination (meta p-value <0.05).

**Conclusion:**

Our results indicate that multiple SNPs from genes involved in cell adhesion, viral attachment, and viral entry, as well as others in genes involved in signaling and/or immune response regulation, play a role in modulating humoral immune responses following live rubella vaccination.

## Introduction

Host genetic determinants play an important role in the generation and regulation of humoral and cellular immune responses after vaccination and/or infection [1], [2]. Studying measles-mumps-rubella (MMR) vaccine immunogenicity in twins, we have previously demonstrated a high level of heritability, approximately 46%, of humoral immune response variance following rubella vaccination [3]. Multiple host genes operate at a gene and gene-network level to shape and regulate the quality, duration and magnitude of rubella vaccine-induced humoral and cellular immune responses [4], [5], [6], [7], [8], [9], [10].

Viral attachment, cell entry, fusion with the cell membrane, and viral genome translocation into target cells are essential early stages initiating the viral infectious cycle. These are key steps in viral replication and dissemination, as well as virus-host interactions involving the generation and maintenance of the immune response. Recent discoveries point to the role of newly discovered cellular receptors and attachment factors for several important human viruses, including rubella virus (Myelin Oligodendrocyte Glycoprotein, MOG), measles virus (measles virus epithelial cell receptor PVRL4, poliovirus receptor-related 4 gene, Nectin-4; and the transmembrane C-type lectin DC-SIGN/CD209), Rift Valley Fever virus/RVFV (DC-SIGN/CD209), poliovirus (Poliovirus receptor, PVR/CD155, Nectin-5) and herpesvirus (poliovirus receptor-related protein 2, PVRL2/CD112, Nectin-2) in infection, disease pathogenesis and immunity [11], [12], [13], [14], [15], [16], [17], [18], [19], [20]. Host cell surface receptors and attachment factors are plausible genetic regulators of measles vaccine-induced immunity [21], [22], [23], [24], but their influence on immunity after rubella vaccination has never been examined.

In the current candidate gene association study, we follow up on previously found genetic associations and also make use of recent major discoveries in the virology field, and explore the plausible role of selected cell surface receptor-, and attachment factor-related genes, such as MOG and poliovirus receptor-related gene family members, in the genetic control of immune response variations after live rubella vaccination. Our results from two independent study cohorts (discovery and replication) strongly suggest that genetic variants from these genes play a role in modulating humoral immune responses following rubella vaccination.

## Methods

The methods described herein are similar or identical to those published for our previous studies [6], [7], [8], [9], [25], [26], [27].

### Study Participants

The study cohort was a large population-based sample of 2,250 healthy children, older adolescents, and healthy adults (age 11 to 40 years), residing in Rochester, MN, and San Diego, CA, with clinical and demographic characteristics previously reported [7], [9], [21], [28], [29], [30].

The Rochester cohort comprised a sample of 1,145 individuals from three independent age-stratified random cohorts of healthy schoolchildren and young adults from all socioeconomic strata from Olmsted County, MN, enrolled between 2001 and 2009, as published elsewhere [7], [9], [21], [28], [29], [30]. Eleven hundred and one parents agreed to let their children join the current rubella vaccine study.

Between July 2005 and September 2006, we enrolled an additional 1,076 healthy older adolescents and healthy adults (age 18 to 40 years, San Diego cohort) from armed forces personnel at the Naval Health Research Center (NHRC) in San Diego, CA, as previously described [30]. All subjects included in the current rubella vaccine study had a documented receipt of measles-mumps-rubella (MMR) vaccine. The Institutional Review Boards of the Mayo Clinic and the NHRC approved the study, and written informed consent was obtained from each subject, from the parents of all children who participated in the study, as well as written assent from age-appropriate participants.

### Rubella Virus-specific Neutralization Assay (sICNA)

We used a modified version of the immunocolorimetric-based neutralization method described by Chen et al. [31], optimized to a high-throughput micro-format, to measure rubella virus-specific neutralizing antibodies [25], [26]. Subjects’ sera were heat-inactivated for 1 hour at 56°C. Sera were serially diluted in two-fold, in triplicate for each dilution, beginning from 1∶12.5 through 1∶100 (to yield a final volume of 30 µL per dilution), using phosphate-buffered saline (PBS, pH 7.4) supplemented with 1% fetal bovine serum (FBS). Rubella virus stock (vaccine virus HPV77) was diluted to a working concentration of 1.2×10^3^ plaque-forming units (PFU)/mL, and was added (30 µL) to an equal volume of diluted serum (or diluent as in the case of virus-only control), yielding a final serum dilution series of 1∶25 through 1∶200. The plate was incubated for 1.5 hour at 37°C, 5% CO_2_. Fifty microliters of each mixture were used to inoculate confluent Vero cell monolayers (cultured in flat-bottom 96-well plates) and the cells were incubated for 1 hour at 37°C, 5% CO_2_. After the incubation period, DMEM supplemented with 5% FBS and 50 µg/mL Gentamicin (Gibco; Invitrogen, CA, USA) was added to each well and the plate was further incubated for 72 hours at 37°C, 5% CO_2_. The plates were developed using an indirect immunocolorimetric method and rubella E1-specific monoclonal antibody (Centers for Disease Control and Prevention/CDC, GA, USA), as previously described [31]. Optical density (OD) values were measured using a measurement/reference wavelength pair of 450 nm/630 nm. The neutralization titer was calculated as the highest dilution at which the input virus signal was reduced by at least 50% within the dilution series (neutralization titer 50/NT_50_). The Loess method of statistical interpolation was used to estimate interpolated NT_50_ values from observed values [25], [32]. The intra-class correlation coefficient (ICC), based on log-transformed estimates from samples with repeated NT_50_ measurements was, 0.89, demonstrating a high degree of reproducibility in the assay [25], [26].

### SNP Selection and Candidate SNP Genotyping

The SNP selection and genotyping methods described herein are similar or identical to those published for our previous genetic association studies [7], [8], [9], [28], [29]. The genotyping effort included follow-up on 197 previously identified genetic associations (p<0.05) between SNPs in immune response genes (cytokine and cytokine receptor genes, Toll-like receptor and signaling genes, vitamin D and vitamin A receptor family genes, antiviral effector and other innate genes) and immune measures after rubella vaccination. A total of 571 additional SNPs on the GoldenGate 768-plex panel were filled with candidate tagSNPs selected from eight newly discovered viral receptor genes (*MOG*, *PVR*, *PVRL1*, *PVRL2*, *PVRL3*, *PVRL4, BTN2A1*, *BTN3A1*) using the tagSNP selection approach based on linkage disequilibrium (LD), as previously described [7], [8], [9], [28], [29], [33].

The 768 SNPs were genotyped using a custom designed 768-plex Illumina GoldenGate™ assay (Illumina Inc., San Diego, CA) following the manufacturer’s instructions. The BeadStudio 2 software was used to call genotypes. The genotyping quality was high, with a mean SNP call rate of 99.7% and 99.8% (for the Rochester cohort and the San Diego cohort, respectively), and a mean subject call rate of 99.7% and 99.8%. The estimated concordance of duplicates was 96%–100%.

For the Rochester cohort, after removing samples that failed genotyping and/or QC checks, there were 1,039 samples and 606 SNPs available for analysis, including 887 samples and 555 SNPs for the Caucasian-only analysis. For the San Diego cohort, we had data (available for analysis) on 989 subjects and 611 SNPs, including 542 samples and 565 SNPs for the Caucasian-only analysis.

### Statistical Methods/Analysis

Summaries of the subject characteristics were obtained within the two study cohorts, counts and percentages for categorical features and medians and interquartile ranges (25th and 75th percentiles) for continuous features. Principal components analysis of a large collection of independent SNPs was applied to estimate and define racial groups, as previously described [34], [35]. As the Rochester cohort was predominantly Caucasian, the data for both cohorts was subset to this race. Hardy–Weinberg equilibrium (HWE) was tested within the Caucasian subsets of each cohort. Principal component analysis was repeated via EIGENSTRAT [36] on a collection of SNPs except those that were in low linkage disequilibrium (r^2^<0.1) and that had HWE p-values>1×10^−3^. Non-autosomal SNPs, or SNPs that had a minor allele frequency less than 0.01, were not included in these population stratification analyses, which produced additional race and cohort-specific population stratification eigenvectors.

Linear regression models, which modeled the quantitative neutralizing antibody outcome as a linear combination of covariates and SNP genotype variables, were used to assess associations between neutralizing antibody levels and SNP genotypes. An additive genetic model, which coded SNP genotypes into a single variable reflecting the number of minor alleles carried by each individual, was used for the primary tests of significance. As sensitivity analyses, dominant and recessive genetic effects were also examined for their associations with the outcome, but for most of the SNPs these alternative models did not prove to be superior compared to the additive genetic model used in our primary assessment of significance. Neutralizing antibody (NT_50_) levels were analyzed as the phenotype of interest after logarithmic transformation in order to conform to linear models assumptions. SNP associations were assessed while adjusting for gender, assay batch effect, quartiles of age at enrollment, immunization age, time since last immunization to enrollment, and race-specific population stratification eigenvectors.

Associations were tested independently within each cohort, and the results were compared between the two cohorts. In order to determine which SNPs were potentially associated with neutralizing antibody titers across the two study cohorts, a fixed effects meta-analysis was performed for each SNP [37]. These meta-analyses used estimates of effect from the two study cohorts, and combined them using a fixed effects paradigm, to obtain a pooled estimate of effect, along with its pooled standard error and test of significance. Additionally, the meta-analyses provided a test of heterogeneity between cohorts. SNPs that did not display significant heterogeneity between the two cohorts (p>0.1) and that had meta-analysis p-values less than 0.05 were considered to be potentially associated with neutralizing antibody levels, and meriting further follow-up in future studies.

Analyses were carried out using the SAS software system (SAS Institute, Inc., Cary, NC) and the PLINK whole genome data analysis software package.

## Results

### Demographic Characteristics and Immune Variables of the Study Subjects

The demographic characteristics of the study cohorts have been reported previously [7], [9], [25], [26], [27], [28], [29], [30], [38], and are summarized in Table 1. Briefly, out of the Rochester (discovery) cohort, 1,029 subjects met all inclusion, exclusion and QC criteria, and had genotyping and rubella neutralizing antibody data available for this study. Of these 1,029 subjects, 887 (85.4%) were Caucasians, and were included in the final analysis. Out of the San Diego (replication) cohort, 985 subjects met all inclusion, exclusion and QC criteria, and had genotyping and rubella neutralizing antibody data available. Of these 985 subjects, 542 (55.03%) were Caucasians, and were included in the final analysis. The median age at enrollment was 15 years (IQR, 13, 17) for the Rochester cohort, and 23 years (IQR, 22, 27) for the San Diego cohort. The median neutralizing antibody level was 55.5 NT_50_ for the Rochester cohort and 62.8 NT_50_ for the San Diego cohort (Table 1) Detailed characterization of the immune response variables in the study cohorts and their interrelationships has been published previously [25], [26], [27].

**Table 1:** Demographic and immunological characteristics of the study subjects included in the analysis.

	Rochester cohort (n = 887)	San Diego cohort (n = 542)	Total (n = 1,429)
**Age at Enrollment**			
Median, IQR^a^ (Years)	15 (13, 17)	23 (22, 27)	17 (14, 22)
**Age at Last Vaccination** (Years)			
Median, IQR^a^	10 (5, 12)	19 (18, 21)	12 (5, 18)
**Time from Last Vaccination to Enrollment**			
Median, IQR^a^ (Years)	6.4 (4.6, 8.5)	3.0 (2.2, 3.9)	5.2 (3.1, 7.5)
**Gender (N, %)**			
Male	487 (54.9%)	394 (72.7%)	881 (61.7%)
Female	400 (45.1%)	148 (27.3%)	548 (38.3%)
**Race** (**N, %**)			
Caucasian	887 (100%)	542 (100%)	1,429 (100%)
**Neutralizing Antibodies** (NT_50_)			
Median, IQR^a^	55.5 (34.4, 91.3)	62.8 (42.3, 108.3)	58.4 (36.5, 96.6)
Mean, SD	72.0 (60.9)	94.6 (127.2)	80.6 (92.7)
Range	17.0–650.8	15.6–1298.0	15.6–1298.0

a IQR, inter-quartile range with 25% and 75% quartiles.

Humoral response is defined as the rubella-specific NT_50_ titer in the sICNA assay. Values reported are in NT_50_ for antibody responses ± IQR/inter-quartile range).

### Associations between SNPs and Antibody Titers in Two Distinct Cohorts

**Figure 1:**
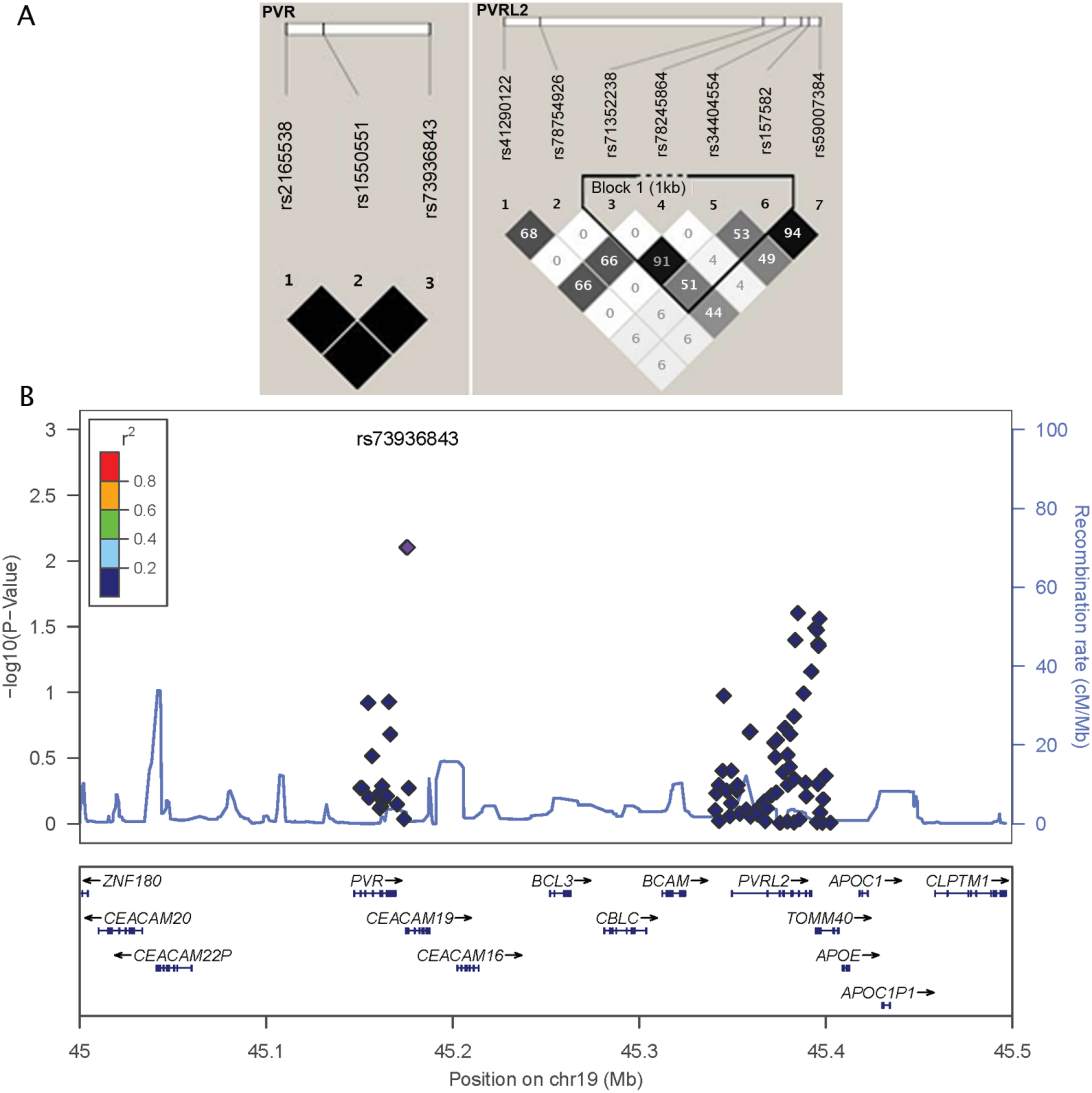
Locus zoom plot and schematic LD block structure of the *PVR* and *PVRL2* genetic variants of interest. A. Haplotype block structure of the *PVR* and *PVRL2* genetic variants, analyzed using Haploview software, version 4.2 (all SNPs presented were directly genotyped in the study). The r^2^ color scheme is: white (r^2^ = 0), shades of grey (0<r^2^<1), black (r^2^ = 1). The numbers report the r^2^ value multiplied by 100. B. Locus zoom plot of PVR/PVRL2 genetic region on 19q13, showing multiple SNPs associated with rubella-specific neutralizing antibody levels. P-value is represented on the left Y-axis. The recombination rate is represented on the right Y-axis and as a line at the bottom of the graph. SNPs are represented as circles, color-coded for LD with respect to rs73936843 (the top three PVR SNPs rs73936843, rs1550551 and rs2165538 are in perfect LD and overlap, as represented on the graph).

We found three replicated genetic associations between SNPs tagging the poliovirus receptor gene (*PVR*, nectin-like protein 5, CD155) in high linkage disequilibrium/LD (rs1550551, rs2165538 and rs73936843; r^2^ = 1, Fig. 1), and rubella-specific neutralizing antibodies (meta-analysis p-value = 0.008, Table 2). Although relatively rare, the heterozygous genotype of these *PVR* SNPs (no homozygous minor allele genotype was observed) was consistently associated with a significant decrease (> 2-fold) in neutralizing antibody titers after rubella vaccination in both cohorts. Similarly, the heterozygous genotype of two SNPs (rs78245864 and rs41290122, in LD; r^2^ = 0.66, Fig. 1) in the *PVRL2* gene (poliovirus receptor-related 2, herpesvirus entry mediator B, nectin 2, CD112) was associated with a significant increase (∼ 30%) in rubella-specific neutralizing antibody titers (meta-analysis p-values of 0.038 and 0.04 for rs78245864 and rs41290122, respectively, Table 2). Furthermore, five additional *PVRL2* genetic variants (some in LD, Fig. 1) demonstrated some evidence for association with rubella-specific NT_50_ titers post vaccination (meta-analysis p-value<0.044, Table 2), although some of the findings (association with high/low response) were not consistent between the two cohorts. Our meta-analysis also revealed potential associations between SNPs in the promoter regions of *CD209/DC-SIGN* (rs2287886) and *IL6* (rs1880241) genes and rubella-specific neutralizing antibodies (meta-analysis p-values of 0.008 and 0.027, for rs2287886 and rs1880241, respectively, Table 2). We found a replicated genetic association for a SNP (rs1153600) in intron 3 of the vitamin A receptor gene *RARB* that demonstrated a significant decrease in neutralizing antibody titers with the representation of the minor allele (meta-analysis p-value = 0.037, Table 2). The homozygous minor allele genotype of a SNP (rs16895223) in the putative rubella virus receptor gene *MOG* (myelin oligodendrocyte glycoprotein) was associated with a decrease in rubella-specific antibodies (meta-analysis p-value = 0.046, Table 2). In addition, *BTN2A1* SNP rs1977198, *IRF9* SNP rs17256713 and *EIF2AK2* SNPrs4648212 also demonstrated evidence for association with rubella-specific NT_50_ titers (p<0.049, Table 2).

**Table 2:** SNPs associated with rubella virus-specific neutralizing antibody responses in Caucasians.

				*ROCHESTER* *COHORT*				*SAN* *DIEGO* *COHORT*			*META* *ANALYSIS*	
Gene	SNP ID/ Location/Function	Geno type^a^	N^a^	Median Ab level NT50 (IQR)^b^	p-value^c^	Geno type^d^	N^d^	Median Ab level NT50 (IQR)^e^	p-value^f^	Meta p-value^g^	Pooled estimate^g^	Homogeneity p-value^g^
*PVR*	rs1550551	AA	878	55.7 (34.5,91.3)	0.037	AA	540	62.9 (42.6,108.8)	0.085	0.008	−0.998	0.606
	Downstream	AG	1	17 (17,17)		AG	2	30.6 (27.6,33.5)				
		GG	0	(−)		GG	0	(−)				
*PVR*	rs2165538	CC	878	55.7 (34.5,91.3)	0.037	CC	540	62.9 (42.6,108.8)	0.085	0.008	−0.998	0.606
	Downstream	CG	1	17 (17,17)		CG	2	30.6 (27.6,33.5)				
		GG	0	(−)		GG	0	(−)				
*PVR*	rs73936843	GG	877	55.5 (34.5,91)	0.037	GG	540	62.9 (42.6,108.8)	0.085	0.008	−0.998	0.605
	Downstream	GA	1	17 (17,17)		GA	2	30.6 (27.6,33.5)				
		AA	0	(−)		AA	0	(−)				
*CD209/*	rs2287886	GG	336	53.6 (33.4,85.8)	0.01	GG	208	61.2 (39.8,105)	0.347	0.008	0.064	0.489
*DC-SIGN*	Promoter	GA	414	55.2 (34.4,91.4)		GA	248	63.2 (43.9,109.6)				
		AA	128	63.2 (36.8,112.6)		AA	85	61.1 (44.9,115.8)				
*PVRL2*	rs78754926	GG	852	55.4 (34.4,90.3)	0.014	GG	529	62.9 (42.2,108.3)	0.727	0.025	0.244	0.283
	Intron	GA	21	76 (52.9,116)		GA	13	57 (44.9,105.7) (−)				
		AA	0	(−)		AA	0					
*IL6*	rs1880241	AA	224	58.7 (36.5,96.3)	0.067	AA	141	63.3 (44.4,112.9)	0.213	0.027	−0.053	0.995
	Promoter	AG	443	56.6 (35.4,90.8)		AG	277	62.2 (42.2,105.4)				
		GG	210	48.6 (30.5,78.1)		GG	124	62.3 (39.6,113.8)				
*BTN2A1*	rs1977198	CC	245	54.7 (34.1,90.6)	0.036	AA	145	65.2 (42.6,114.5)	0.391	0.027	−0.051	0.649
	Intron	CA	421	53.1 (34,88.6)		CA	263	59.9 (42.7,105.6)				
	(boundary)	AA	213	61.5 (39.5,95)		CC	134	63.6 (41.3,109.3)				
*PVRL2*	rs59007384	CC	534	54 (33.2,89.7)	0.129	CC	328	63.4 (43.3,105)	0.093	0.028	0.063	0.984
	Downstream	CA	304	59.7 (37,94.8)		CA	189	59.5 (40.7,107)				
		AA	41	57.7 (45.4,96.1)		AA	25	94.6 (65.3,130.2)				
*PVRL2*	rs71352238	AA	638	54.2 (33.9,88.6)	0.076	AA	381	62.7 (41.5,105.3)	0.235	0.032	0.070	0.588
	Downstream	AG	221	64.2 (39.1,104.3)		AG	149	61.1 (44.6,111.2)				
		GG	20	53.7 (47.8,78.7)		GG	12	98.1 (69.3,122.5)				
*RARB*	rs1153600	GG	387	59.5 (34.5,93.8)	0.33	GG	250	65.1 (43.7,110.1)	0.022	0.037	−0.052	0.171
	Intron	GA	386	52.4 (34,84.5)		GA	234	63.4 (42.3,111.1)				
		AA	105	55.4 (36.5,91.3)		AA	58	55.1 (40.7,90.1)				
*PVRL2*	rs78245864	AA	866	55.4 (34.4,90.7)	0.022	AA	535	62.7 (42.3,108.3)	0.736	0.038	0.298	0.349
	Downstream	AC	13	76 (52.9,120)		AC	7	83.8 (40.7,114.6)				
		CC	0	(−)		CC	0	(−)				
*IRF9*	rs17256713	GG	723	56.5 (35.1,92.6)	0.036	GG	456	62.8 (42.3,108.2)	0.608	0.04	−0.085	0.497
	Downstream	GA	148	50.2 (31.4,88.5)		GA	83	62.4 (42.1,108.3)				
		AA	8	36.6 (30.2,67)		AA	3	92.1 (45.9,156.3)				
*PVRL2*	rs41290122	GG	856	55.4 (34.4,90.7)	0.053	GG	532	62.6 (42.2,107)	0.452	0.04	0.224	0.762
	Intron	GA	23	72.4 (52.9,116)		GA	10	94.8 (53.4,122.5)				
		AA	0	(−)		AA	(−)	(−)				
*PVRL2*	rs34404554	GG	642	54.5 (34,89.1)	0.078	GG	382	62.3 (41.5,105.3)	0.313	0.043	0.068	0.881
	Downstream	GC	217	63.3 (38.2,104.3)		GC	148	61.9 (43.7,112.9)				
		CC	19	52.7 (45.4,82.3)		CC	10	88.8 (65.3,113.9)				
*PVRL2*	rs157582	GG	523	54 (32.7,89.1)	0.137	GG	322	64.1 (43.2,105.4)	0.165	0.044	0.057	0.745
	Downstream	GA	309	59.5 (37.7,95)		GA	195	59.4 (40.7,105.8)				
		AA	44	58.6 (45.2,97.6)		AA	25	94.6 (65.3,126.5)				
*MOG*	rs16895223	GG	654	54.7 (34.2,88.4)	0.302	GG	398	63 (42.7,105.3)	0.05	0.046	0.07	0.329
	Intron	GC	211	61.2 (35.9,107.3)		GC	129	60.6 (42.1,119.1)				
		CC	10	34.6 (31.5,44.7)		CC	13	62.4 (46.2,143.9)				
*EIF2AK2*	rs4648212	GG	758	56.7 (34.6,93.6)	0.024	GG	488	63.2 (42.3,110.4)	0.949	0.049	−0.095	0.261
	Intron	GA	105	48.8 (29.6,80.5)		GA	49	57.2 (45.3,103.3)				
		AA	5	40.6 (26.9,48.4)		AA	3	54.3 (21.5,322.1)				

a Genotypes for the Rochester cohort, presented as homozygous major allele/heterozygous/homozygous minor allele. N indicates the number of subjects with the specific genotype.

b Immune outcomes for the Rochester cohort, presented as median neutralizing antibody titer in NT_50_± IQR (inter-quartile range), as measured by the sICNA assay.

c Ordinal p-value for analysis in the Rochester cohort only, after adjusting for gender, assay batch effect, quartiles of age at enrollment, immunization age, time since last immunization to enrollment, and race-specific population stratification eigenvectors.

d Genotypes for the San Diego cohort, presented as homozygous major allele/heterozygous/homozygous minor allele. N indicates the number of subjects with the specific genotype.

e Immune outcomes for the San Diego cohort, presented as median neutralizing antibody titer in NT_50_± IQR (inter-quartile range), as measured by the sICNA assay.

f Ordinal p-value for analysis in the San Diego cohort only, after adjusting for gender, assay batch effect, quartiles of age at enrollment, immunization age, time since last immunization to enrollment, and race-specific population stratification eigenvectors.

g Meta-analysis p-value after adjusting for gender, assay batch effect, quartiles of age at enrollment, immunization age, time since last immunization to enrollment, and race-specific population stratification eigenvectors; pooled estimate from the meta analysis showing the magnitude and direction of the estimated effect on the immune measure; homogeneity test p-value estimating the homogeneity between the two cohorts. Only SNPs with p-value below 0.05 in the meta-analysis and homogeneity test p-value above 0.1 are presented.

The analysis results for all SNPs assessed for association with neutralizing antibody levels in our current study are presented in Table S1.

## Discussion

Much knowledge has been accumulated on the role of genetic factors in the inter-individual immune response variation following immunization with measles-mumps-rubella (MMR) vaccine. A genome-wide transcriptional study (using mRNA-Seq) in high and low antibody responders to rubella vaccine identified HLA genes, and several innate immunity and inflammation-related genes (*MEFV*, Mediterranean fever gene, *EMR3*, EGF-like module containing, mucin-like, hormone receptor-like 3 gene, etc.) that discriminated between high and low humoral immune response following rubella immunization [4]. We and others have highlighted the importance of HLA polymorphisms, SNPs in cytokine and cytokine receptor genes, antiviral genes, toll-like receptors and pathway signaling genes, vitamin A and D receptor and cellular viral receptor genes (*CD46* and *SLAM* for measles virus), and other important innate immune genes (*TRIM* genes, *DC-SIGN*, etc.) for inter-individual immune response variation after measles and rubella vaccination [2], [5], [6], [7], [8], [9], [10], [21], [22], [23], [24], [28], [29], [39], [40], [41], [42]. Because the immune response encompasses a system of interrelated components and biological processes, it is likely that multiple genetic factors and gene networks influence host response and vaccine-induced immunity (involving antibody response), including measles and rubella vaccines [5], [40].

The purpose of the current study was to refine our previous findings through replication, and to examine, in two independent cohorts, the role of newly discovered host viral receptors, attachment factors and immune regulators in the adaptive immune response induction and/or maintenance following vaccination with a live rubella vaccine.

Our meta-analysis results provide evidence for the involvement of multiple genetic variants (some in LD) within the PVR-PVRL2 gene region (Fig. 1) on chromosome 19 (19q13.31–32) in the genetic control of neutralizing antibody response to rubella vaccine. The poliovirus receptor (*PVR*, CD155) and poliovirus receptor-related 2 (*PVRL2*, CD112) genes encode transmembrane glycoproteins/nectins of the immunoglobulin (Ig) superfamily, which are components of adherens junctions and serve as cellular entry receptors for poliovirus (PVR), pseudorabies virus, and certain mutant strains of herpes simplex virus (PRRL2) [17], [18], [19]. As virus-specific cell surface receptors, these proteins have important functional downstream effects on processes related to viral entry, such as viral replication, cell-to-cell spread, viral tropism, pathogenesis and antiviral immunity. For example, the Ala67Thr mutation in the poliovirus receptor gene (*PVR*) was previously associated with a higher risk for developing vaccine-induced and wild-type virus-induced poliomyelitis [43].

Furthermore, both PVR (CD155) and PVRL2 (CD112) are directly involved in immunity and regulate T cell (CTL) and natural killer/NK cell activation/cytotoxicity by functioning as specific ligands for the immune costimulatory receptors DNAX Accessory Molecule-1/DNAM-1 (CD226) and CD96/Tactile and by promoting IFNγ production [15], [44], [45], [46]. Importantly, CD155/PVR may play a role in Th2 differentiation and humoral immunity by polarizing naïve CD4^+^T cells to a Th2 phenotype [16]. In particular, TLR agonists are known to: upregulate CD155/PVR expression (dependent on MYD88, TRIF and NF-κB); suppress Th1 cell differentiation; and modulate antigen/(OVA)-specific IgG titers in a mouse model [16]. Consistent with these findings, Maier *et al.* demonstrated less efficient humoral immune response (IgG and IgA) to orally administered antigens in mice deficient of CD155 [47].

In summary, based on the literature and our findings, we speculate that one or more of the multiple PVR and PVRL2 genetic variants, associated with variation in rubella vaccine-specific neutralizing antibody levels, or other tagged causal SNP/SNPs in the 19q13 region, influence the gene expression and/or protein function of CD155 (*PVR*) and CD112 (*PVRL2*). In turn, this may have downstream effects on rubella virus-specific humoral immunity by either modulating the unspecific (or specific) virus attachment/entry, replication and immune response priming or, more likely, by modulating Th1/Th2 cell differentiation and promoting more (or less) efficient antibody response [16].

The only cellular receptor for rubella virus identified to date is the myelin oligodendrocyte glycoprotein (MOG), an adhesion molecule that is important in nerve myelination and is implicated as a target antigen in the pathogenesis of several autoimmune demyelinating diseases (including multiple sclerosis/MS), and in the central nervous system (CNS) damage in congenital rubella syndrome [11]. However, rubella virus cell tropism and infectivity suggest that other, yet undiscovered, cellular receptors and adhesion factors capable of mediating infection exist for this pathogen. MOG is expressed predominantly in the central nervous system and is hardly detectable in immune system organs/tissues and cells such as spleen, thymus and PBMCs [11]. Our analysis identified two intronic polymorphisms (rs16895223, p = 0.046 and rs1977198, p = 0.027, Table 2) in the *MOG* gene and the highly homologous butyrophilin *BTN2A1* gene, both on chromosome 6p22, that were associated with variations in rubella-specific neutralizing antibody response. Host genetic variation in these or other tagged functional *MOG/BTN2A1* SNPs may potentially impair or enhance viral entry into susceptible cells, alter virus replication/propagation and antigen abundance and modulate subsequent immune response. Alternatively, these genes may interfere in immune regulation, as demonstrated for the butyrophilins. The latter are co-stimulatory molecules that are implicated in T cell inhibition (negative regulation of T cell proliferation, activation, cytokine production), immune signaling and interaction with DC-SIGN on dendritic cells (BTN2A1 is identified as a ligand for DC-SIGN), and genetic variation in these genes have been associated with inflammatory diseases [48], [49].

Of note, our results also demonstrate an association between a promoter SNP in *DC-SIGN* (rs2287886, p = 0.008, Table 2) and humoral immune response variations following rubella vaccination. DC-SIGN (Cell-Specific Intercellular adhesion molecule-3-Grabbing Non-integrin, CD209) is a C-type lectin that mediates dendritic cell function and activation of CD4^+^ T cells and binds multiple microorganisms, including viruses, by recognizing mannose type glycoproteins [20]. This molecule is exploited by many viruses for attachment and/or cellular entry: measles virus (MV); rift valley fever virus (RVFV); influenza A viruses; human immunodeficiency virus (HIV-1); hepatitis C virus (HCV); herpes simplex virus (HSV1); human cytomegalovirus (HCMV); Ebola virus; SARS coronavirus and dengue virus [20]. Polymorphisms in *DC-SIGN* (and, in particular, the promoter SNP rs2287886) have been associated with the clinical course and outcome of dengue virus infection, cytomegalovirus infection, tick-borne encephalitis virus (TBEV) infection and pulmonary aspergillosis [50], [51], [52]. Furthermore, the promoter SNP rs2287886 is functional and was demonstrated to influence *DC-SIGN* gene expression and HCMC infection in dendritic cells [52]. Interestingly, we found the same *DC-SIGN* promoter SNP to be associated with variations in measles vaccine-induced TNFα secretion in African-Americans and several other *DC-SIGN* SNPs that were associated with variations in measles-specific neutralizing antibody levels in Caucasians and African-Americans [21]. Therefore, it is likely that *DC-SIGN, PVR, PVRL2* or other attachment factors and/or cellular viral receptors and pathway-related genes are involved in the genetic control of rubella vaccine-induced immune response heterogeneity after vaccination.

Another important finding is the observed genetic association between a SNP (rs1153600) in intron 3 of the retinoic acid receptor, beta (*RARB*) gene and rubella-specific neutralizing antibody response (the homozygous minor allele and the heterozygous genotypes were associated with lower antibody titers in both cohorts). RARB is a receptor for retinoic acid, the biologically active metabolite of vitamin A, with involvement in cell growth, differentiation, cell signaling and gene regulation. Interestingly, vitamin A has direct effects on the immune system and regulates antigen presentation, lymphocyte homing, proliferation of B and T cells, T-helper differentiation, cytokine production, T cell activation and cytotoxicity, as well as enhancing the antibody response to vaccination, including the measles vaccine, oral polio vaccine, and tetanus and diphtheria toxoids [53], [54]. Furthermore, our previous studies demonstrated associations between: the same *RARB* SNP (rs1153600, p = 0.034) and two other *RARB* SNPs and rubella (whole) virus-specific IgG levels (as measured by a chemiluminescent immunoassay) in 738 children/young adults following two doses of MMR vaccine [8]; and *RARB* SNPs/haplotypes and variations in measles-specific neutralizing antibody levels and cellular immune outcomes after MMR vaccination [41]. The findings from the current study and previous reports add evidence to the importance of vitamin A receptors and pathway in immune response regulation after immunization.

Other potential findings include SNP associations between polymorphisms in the *IL6* gene (promoter region SNP rs1880241), *IRF9* gene (3′intergenic SNP rs17256713) and *EIF2AK2* gene (intronic SNP rs4648212), which we previously found to be associated with whole rubella virus-specific IgG levels using single-SNP and/or multigenic assessment analysis [5], [7]. Other SNP associations identified in our previous studies were not confirmed in the current study, possibly due to differences in study design, sample size and cohorts’ characteristics, analytical approach, differences in immune response measurement (rubella whole virus-specific IgG levels vs. rubella virus-specific neutralizing antibody levels), and/or false-positive findings [4], [5], [6], [7], [8], [9], [10].

While our efforts have identified a collection of genetic variants that are of high interest as potentially playing a role in variability of neutralizing antibody response after rubella vaccination, it must be noted that some of the genetic associations may not be true positives. The limitations of our study reflect the fact that the characteristics of the study participants were different between the two study cohorts. This made it difficult to compare and/or combine the results from the two cohorts using a method that would typically be applied to control for false-positive findings. For this reason, we applied meta-analytic approaches to evaluate the degree of evidence of association present when evaluating combined results across the two cohorts, with the understanding that false-positive results would be less likely to show consistent associations between cohorts. Given the signals that we observed, and the biological plausibility, it is likely that at least several of the identified associations/genes do indeed play a role in influencing neutralizing antibody levels observed in vaccinated individuals. In addition, our analysis focused on additive genetic effects, and while the support for other genetic models over the additive model was not compellingly strong in our sensitivity analyses, it is possible that for some of the genetic variants an alternative genetic model may prove superior. Other limitations include the influence of unpredictable factors confounding analysis results (e.g., potential wild-type rubella virus exposure in deployed military personnel from the San Diego cohort).

The strengths of our study include the use of a clinically relevant immune outcome to assess vaccine response (i.e., rubella-specific functional/neutralizing antibody levels measured using a state-of-the-art, standardized, high-throughput immune assay). A significant strength is the genetic association analysis approach, adjusting for known confounding variables, and the use of two independent cohorts (subsetted to Caucasian subjects only) to minimize the false-positive findings. The identified genes and genetic variants will be further fine mapped in order to identify candidate functional variants in the genomic regions tagged by the original SNP (if not functional). The p-values, and magnitudes of effect, obtained from the fine-mapping analyses will provide important insights into which of the variants is most likely to be causing the genetic association that was originally observed. Finally, the identification of likely causal genetic variants will be complemented with functional studies to reveal the SNP immediate functional effect and the downstream consequences/biological mechanisms for humoral immune response variation.

In summary, our results identified multiple genetic variants, primarily in genes related to viral attachment and entry, and/or immune regulation that are associated with inter-individual differences in rubella-specific neutralizing antibody response after vaccination. Such findings may indicate evidence for novel receptors used by the rubella virus for cell entry, and will assist in development of improved vaccines and vaccine formulations for achieving optimal immune response after vaccination.

## Supporting Information

Table S1 **SNPs assessed for association with neutralizing antibody levels after rubella vaccination in two different cohorts.**
(DOCX)
