# Supplementary material for: Associations between Single Nucleotide Polymorphisms in Cellular Viral Receptors and Attachment Factor-Related Genes and Humoral Immunity to Rubella Vaccination
Source: PLoS One. 2014 Jun 19;9(6):e99997. doi: 10.1371/journal.pone.0099997 (PMC4063777; doi:10.1371/journal.pone.0099997)
Supplement: Table S1 — SNPs assessed for association with neutralizing antibody levels after rubella vaccination in two different cohorts. (DOCX) [file pone.0099997.s001.docx]

**Table S1**. SNPs assessed for association with neutralizing antibody levels after rubella vaccination in two different cohorts

|  | | | **Rochester cohort** | | | | **San Diego cohort** | | | | **Pooled Results** | | | |
| --- | --- | --- | --- | --- | --- | --- | --- | --- | --- | --- | --- | --- | --- | --- |
| **SNP ID**^a^ | **Chr**^a^**.** | **Position**^a^ | **p-value**^b^ | **Estimate**^c^ | **SE**^c^ | **MAF (%)**^d^ | **p-value**^e^ | **Estimate**^f^ | **SE**^f^ | **MAF (%)**^g^ | **Estimate**^h^ | **SE**^h^ | **Homogeneity p-value**^i^ | **Meta p-value**^j^ |
| rs2256774 | 10 | 6097165 | 7.40E-06 | 0.130 | 0.029 | 35.2 | 6.76E-01 | -0.018 | 0.044 | 35.1 | 0.085 | 0.024 | 0.0045 | 4.20E-04 |
| rs2287886 | 19 | 7812536 | 1.01E-02 | 0.075 | 0.029 | 38.1 | 3.47E-01 | 0.040 | 0.042 | 38.6 | 0.064 | 0.024 | 0.48875 | 7.88E-03 |
| rs1550551 | 19 | 45175316 | 3.74E-02 | -1.235 | 0.592 | 0.1 | 8.51E-02 | -0.839 | 0.486 | 0.2 | -0.998 | 0.376 | 0.60547 | 7.90E-03 |
| rs2165538 | 19 | 45175131 | 3.74E-02 | -1.235 | 0.592 | 0.1 | 8.51E-02 | -0.839 | 0.486 | 0.2 | -0.998 | 0.376 | 0.60547 | 7.90E-03 |
| rs73936843 | 19 | 45175842 | 3.74E-02 | -1.235 | 0.592 | 0.1 | 8.51E-02 | -0.839 | 0.486 | 0.2 | -0.998 | 0.376 | 0.6051 | 7.90E-03 |
| rs78754926 | 19 | 45385102 | 1.40E-02 | 0.322 | 0.131 | 1.2 | 7.27E-01 | 0.069 | 0.196 | 1.2 | 0.244 | 0.109 | 0.28275 | 2.49E-02 |
| rs1977198 | 6 | 26466225 | 3.64E-02 | 0.058 | 0.028 | 48.4 | 3.91E-01 | -0.036 | 0.041 | 49.0 | -0.051 | 0.023 | 0.64865 | 2.65E-02 |
| rs1880241 | 7 | 22759469 | 6.69E-02 | -0.052 | 0.029 | 49.2 | 2.13E-01 | -0.053 | 0.042 | 48.4 | -0.053 | 0.024 | 0.99474 | 2.66E-02 |
| rs59007384 | 19 | 45396665 | 1.29E-01 | 0.052 | 0.034 | 21.9 | 9.25E-02 | 0.085 | 0.051 | 22.0 | 0.063 | 0.028 | 0.58844 | 2.76E-02 |
| rs71352238 | 19 | 45394336 | 7.56E-02 | 0.071 | 0.040 | 14.8 | 2.35E-01 | 0.069 | 0.058 | 16.0 | 0.070 | 0.033 | 0.98401 | 3.23E-02 |
| rs78245864 | 19 | 45395193 | 2.19E-02 | 0.381 | 0.166 | 0.7 | 7.36E-01 | 0.089 | 0.264 | 0.6 | 0.298 | 0.140 | 0.34915 | 3.37E-02 |
| rs1153600 | 3 | 25569565 | 3.30E-01 | -0.029 | 0.030 | 34.0 | 2.22E-02 | -0.102 | 0.045 | 32.3 | -0.052 | 0.025 | 0.17132 | 3.73E-02 |
| rs17256713 | 14 | 24639699 | 3.57E-02 | -0.102 | 0.048 | 9.3 | 6.08E-01 | -0.040 | 0.077 | 8.2 | -0.084 | 0.041 | 0.49678 | 3.97E-02 |
| rs41290122 | 19 | 45383583 | 5.33E-02 | 0.243 | 0.126 | 1.4 | 4.52E-01 | 0.166 | 0.221 | 0.9 | 0.224 | 0.109 | 0.76226 | 4.00E-02 |
| rs34404554 | 19 | 45395909 | 7.80E-02 | 0.071 | 0.040 | 14.5 | 3.13E-01 | 0.060 | 0.060 | 15.6 | 0.068 | 0.033 | 0.88147 | 4.26E-02 |
| rs157582 | 19 | 45396219 | 1.37E-01 | 0.051 | 0.034 | 22.6 | 1.65E-01 | 0.070 | 0.051 | 22.6 | 0.057 | 0.028 | 0.74525 | 4.43E-02 |
| rs16895223 | 6 | 29626949 | 3.02E-01 | 0.045 | 0.044 | 13.1 | 4.99E-02 | 0.117 | 0.059 | 14.4 | 0.070 | 0.035 | 0.32877 | 4.62E-02 |
| rs669260 | 9 | 32503440 | 7.48E-03 | 0.106 | 0.039 | 15.1 | 7.04E-01 | -0.021 | 0.056 | 15.9 | 0.064 | 0.032 | 0.06469 | 4.78E-02 |
| rs4648212 | 2 | 37353011 | 2.39E-02 | -0.128 | 0.057 | 6.6 | 9.49E-01 | -0.006 | 0.093 | 5.1 | -0.095 | 0.048 | 0.26064 | 4.94E-02 |
| rs7648325 | 3 | 25459497 | 8.31E-02 | -0.052 | 0.030 | 42.6 | 3.68E-01 | -0.038 | 0.042 | 39.6 | -0.047 | 0.024 | 0.78375 | 5.29E-02 |
| rs29254 | 6 | 29623594 | 1.65E-01 | 0.093 | 0.067 | 5.0 | 1.65E-01 | 0.133 | 0.096 | 4.9 | 0.106 | 0.055 | 0.72812 | 5.31E-02 |
| rs3824949 | 11 | 5701409 | 5.74E-02 | 0.055 | 0.029 | 44.0 | 5.60E-01 | 0.025 | 0.043 | 44.6 | 0.046 | 0.024 | 0.55649 | 5.73E-02 |
| rs385492 | 6 | 29649547 | 2.54E-01 | -0.032 | 0.028 | 47.0 | 1.06E-01 | -0.068 | 0.042 | 43.5 | -0.043 | 0.023 | 0.47764 | 6.43E-02 |
| rs6857 | 19 | 45392254 | 1.63E-01 | 0.053 | 0.038 | 16.9 | 2.42E-01 | 0.063 | 0.054 | 18.9 | 0.056 | 0.031 | 0.88428 | 6.96E-02 |
| rs2243263 | 5 | 132013299 | 4.30E-01 | 0.036 | 0.045 | 11.4 | 4.03E-02 | 0.148 | 0.072 | 8.9 | 0.068 | 0.038 | 0.18834 | 7.73E-02 |
| rs7910961 | 10 | 6077796 | 3.62E-02 | 0.061 | 0.029 | 35.5 | 9.78E-01 | 0.001 | 0.044 | 35.6 | 0.043 | 0.024 | 0.25195 | 7.88E-02 |
| rs3870968 | 6 | 29647149 | 2.17E-01 | 0.079 | 0.064 | 5.4 | 1.92E-01 | 0.124 | 0.095 | 5.0 | 0.093 | 0.053 | 0.69346 | 7.93E-02 |
| rs10492028 | 12 | 113337163 | 3.97E-01 | 0.029 | 0.034 | 23.2 | 6.73E-02 | 0.093 | 0.051 | 20.8 | 0.049 | 0.028 | 0.2941 | 8.43E-02 |
| rs10774669 | 12 | 113339427 | 3.26E-01 | 0.033 | 0.034 | 23.6 | 1.17E-01 | 0.078 | 0.050 | 21.8 | 0.048 | 0.028 | 0.45929 | 8.96E-02 |
| rs2069778 | 4 | 123376135 | 7.13E-01 | 0.014 | 0.039 | 16.2 | 1.12E-02 | 0.151 | 0.060 | 16.7 | 0.055 | 0.032 | 0.05324 | 9.02E-02 |
| rs6916321 | 6 | 26464789 | 1.19E-01 | 0.053 | 0.034 | 23.0 | 4.74E-01 | 0.035 | 0.049 | 22.8 | 0.047 | 0.028 | 0.77159 | 9.02E-02 |
| rs10410342 | 19 | 7808452 | 1.74E-01 | -0.087 | 0.064 | 5.0 | 3.44E-01 | -0.099 | 0.104 | 4.2 | -0.090 | 0.054 | 0.92488 | 9.77E-02 |
| rs11536889 | 9 | 120478131 | 2.87E-01 | 0.043 | 0.041 | 14.7 | 1.84E-01 | 0.076 | 0.057 | 15.4 | 0.054 | 0.033 | 0.64464 | 1.01E-01 |
| rs34342646 | 19 | 45388130 | 2.25E-01 | 0.048 | 0.039 | 15.3 | 2.62E-01 | 0.065 | 0.058 | 16.1 | 0.053 | 0.033 | 0.79962 | 1.02E-01 |
| rs61642202 | 19 | 45345283 | 2.75E-01 | -0.197 | 0.180 | 0.6 | 2.34E-01 | -0.215 | 0.181 | 1.4 | -0.206 | 0.128 | 0.94137 | 1.06E-01 |
| rs4833248 | 4 | 123380405 | 5.03E-02 | 0.064 | 0.032 | 27.1 | 9.89E-01 | -0.001 | 0.048 | 27.4 | 0.043 | 0.027 | 0.26534 | 1.07E-01 |
| rs17882988 | 1 | 12242870 | 3.93E-01 | 0.033 | 0.039 | 17.0 | 1.01E-01 | 0.103 | 0.063 | 14.3 | 0.052 | 0.033 | 0.3419 | 1.12E-01 |
| rs3755276 | 2 | 102978459 | 2.16E-01 | -0.036 | 0.029 | 37.5 | 3.30E-01 | -0.044 | 0.045 | 34.4 | -0.039 | 0.025 | 0.89093 | 1.16E-01 |
| rs445150 | 6 | 29646879 | 1.13E-01 | 0.050 | 0.032 | 30.7 | 6.34E-01 | 0.021 | 0.045 | 31.0 | 0.041 | 0.026 | 0.59934 | 1.17E-01 |
| rs714948 | 19 | 45165912 | 6.65E-02 | -0.086 | 0.047 | 9.8 | 9.08E-01 | -0.008 | 0.066 | 11.2 | -0.060 | 0.038 | 0.33088 | 1.18E-01 |
| rs1233367 | 6 | 29622220 | 2.00E-01 | 0.039 | 0.030 | 32.6 | 3.76E-01 | 0.039 | 0.044 | 35.0 | 0.039 | 0.025 | 0.99494 | 1.19E-01 |
| rs17714931 | 19 | 45154614 | 3.16E-01 | -0.081 | 0.080 | 3.6 | 2.20E-01 | -0.120 | 0.098 | 4.6 | -0.097 | 0.062 | 0.75249 | 1.20E-01 |
| rs16891666 | 6 | 26397855 | 2.59E-02 | 0.442 | 0.198 | 0.6 | 7.82E-01 | 0.040 | 0.145 | 2.1 | 0.180 | 0.117 | 0.10118 | 1.24E-01 |
| rs2256266 | 6 | 29632318 | 5.69E-01 | -0.020 | 0.035 | 21.9 | 6.46E-02 | -0.092 | 0.050 | 21.6 | -0.044 | 0.029 | 0.23838 | 1.24E-01 |
| rs10176664 | 2 | 102976172 | 2.42E-01 | -0.034 | 0.029 | 37.6 | 3.30E-01 | -0.044 | 0.045 | 34.4 | -0.037 | 0.025 | 0.86241 | 1.30E-01 |
| rs6456720 | 6 | 26414417 | 1.08E-01 | 0.210 | 0.131 | 1.2 | 6.05E-01 | 0.068 | 0.131 | 2.7 | 0.139 | 0.093 | 0.44142 | 1.32E-01 |
| rs6927012 | 6 | 26471992 | 6.78E-02 | 0.083 | 0.045 | 10.8 | 9.91E-01 | 0.001 | 0.064 | 11.5 | 0.056 | 0.037 | 0.29606 | 1.33E-01 |
| rs2069762 | 4 | 123377980 | 7.88E-02 | 0.057 | 0.032 | 27.3 | 9.27E-01 | 0.004 | 0.047 | 28.0 | 0.040 | 0.027 | 0.35596 | 1.33E-01 |
| rs439812 | 6 | 29616607 | 5.78E-01 | -0.019 | 0.035 | 22.3 | 7.58E-02 | -0.088 | 0.050 | 21.9 | -0.042 | 0.029 | 0.25772 | 1.38E-01 |
| rs627928 | 1 | 182551337 | 1.11E-01 | -0.045 | 0.028 | 44.4 | 7.99E-01 | -0.011 | 0.043 | 46.5 | -0.035 | 0.024 | 0.50669 | 1.41E-01 |
| rs1557865 | 12 | 113342598 | 6.81E-01 | 0.015 | 0.036 | 20.1 | 4.40E-02 | 0.108 | 0.054 | 18.2 | 0.044 | 0.030 | 0.14912 | 1.41E-01 |
| rs6910921 | 6 | 26471295 | 1.25E-03 | 0.445 | 0.137 | 1.1 | 5.88E-02 | -0.345 | 0.182 | 1.5 | 0.159 | 0.110 | 0.00054 | 1.48E-01 |
| rs9467761 | 6 | 26467325 | 1.25E-03 | 0.445 | 0.137 | 1.1 | 5.88E-02 | -0.345 | 0.182 | 1.5 | 0.159 | 0.110 | 0.00054 | 1.48E-01 |
| rs12420760 | 11 | 119559464 | 1.47E-01 | -0.083 | 0.057 | 7.2 | 6.77E-01 | -0.036 | 0.086 | 6.6 | -0.068 | 0.048 | 0.64621 | 1.50E-01 |
| rs3024622 | 16 | 27365453 | 9.95E-01 | 0.000 | 0.030 | 33.7 | 1.31E-02 | 0.106 | 0.043 | 37.2 | 0.035 | 0.025 | 0.04221 | 1.50E-01 |
| rs10790329 | 11 | 119537683 | 1.65E-01 | -0.044 | 0.032 | 26.0 | 6.12E-01 | -0.024 | 0.048 | 26.3 | -0.038 | 0.027 | 0.72614 | 1.51E-01 |
| rs11669338 | 19 | 45382984 | 4.58E-01 | -0.034 | 0.046 | 10.3 | 1.38E-01 | -0.106 | 0.071 | 9.8 | -0.055 | 0.039 | 0.39638 | 1.53E-01 |
| rs12814926 | 12 | 113424475 | 3.31E-01 | 0.033 | 0.033 | 22.2 | 2.86E-01 | 0.054 | 0.051 | 20.6 | 0.039 | 0.028 | 0.72107 | 1.61E-01 |
| rs1796524 | 6 | 26449219 | 5.99E-02 | 0.084 | 0.044 | 11.4 | 7.82E-01 | -0.018 | 0.064 | 11.8 | 0.051 | 0.037 | 0.19326 | 1.64E-01 |
| rs5745993 | 1 | 12247008 | 4.40E-01 | 0.029 | 0.038 | 17.0 | 1.72E-01 | 0.081 | 0.059 | 14.9 | 0.044 | 0.032 | 0.46091 | 1.65E-01 |
| rs2535249 | 6 | 29634617 | 4.33E-01 | 0.067 | 0.086 | 2.9 | 2.21E-01 | 0.127 | 0.104 | 4.3 | 0.092 | 0.066 | 0.65928 | 1.66E-01 |
| rs2010604 | 12 | 113408208 | 3.82E-01 | 0.027 | 0.031 | 31.4 | 2.53E-01 | 0.051 | 0.045 | 32.7 | 0.035 | 0.026 | 0.65973 | 1.70E-01 |
| rs2834164 | 21 | 34621948 | 3.06E-01 | -0.029 | 0.028 | 48.9 | 3.62E-01 | -0.038 | 0.041 | 49.7 | -0.032 | 0.023 | 0.85872 | 1.74E-01 |
| rs34278513 | 19 | 45378144 | 1.93E-01 | 0.058 | 0.045 | 11.0 | 6.66E-01 | 0.029 | 0.067 | 10.5 | 0.049 | 0.037 | 0.71588 | 1.86E-01 |
| rs72937915 | 3 | 110839526 | 1.70E-01 | 0.114 | 0.083 | 3.0 | 7.48E-01 | 0.036 | 0.113 | 3.7 | 0.087 | 0.067 | 0.57822 | 1.95E-01 |
| rs13433394 | 21 | 42831910 | 2.69E-01 | -0.043 | 0.039 | 16.1 | 5.00E-01 | -0.039 | 0.058 | 15.3 | -0.042 | 0.032 | 0.95993 | 1.95E-01 |
| rs626214 | 9 | 32532469 | 7.65E-03 | -0.076 | 0.028 | 49.4 | 7.90E-02 | 0.077 | 0.044 | 48.5 | -0.031 | 0.024 | 0.00339 | 1.98E-01 |
| rs3112438 | 19 | 45359570 | 6.01E-01 | -0.018 | 0.034 | 24.1 | 1.32E-01 | -0.074 | 0.049 | 24.6 | -0.035 | 0.028 | 0.34344 | 1.99E-01 |
| rs5746059 | 1 | 12262792 | 5.50E-01 | 0.020 | 0.033 | 24.1 | 1.50E-01 | 0.073 | 0.051 | 21.8 | 0.035 | 0.027 | 0.37422 | 2.00E-01 |
| rs2927466 | 19 | 45359148 | 5.43E-01 | -0.020 | 0.033 | 24.1 | 1.71E-01 | -0.067 | 0.049 | 24.7 | -0.035 | 0.028 | 0.43117 | 2.02E-01 |
| rs11966630 | 6 | 26399893 | 3.83E-02 | 0.293 | 0.141 | 1.1 | 7.79E-01 | -0.040 | 0.142 | 2.2 | 0.127 | 0.100 | 0.0964 | 2.03E-01 |
| rs1041867 | 21 | 34727252 | 7.90E-01 | 0.008 | 0.029 | 44.5 | 1.00E-02 | -0.107 | 0.042 | 43.8 | -0.030 | 0.024 | 0.02342 | 2.04E-01 |
| rs2273558 | 6 | 26466035 | 2.60E-01 | 0.035 | 0.031 | 29.0 | 5.53E-01 | 0.027 | 0.046 | 31.5 | 0.032 | 0.025 | 0.89299 | 2.05E-01 |
| rs73035576 | 19 | 45166466 | 1.63E-01 | -0.077 | 0.055 | 7.3 | 9.13E-01 | -0.010 | 0.094 | 5.7 | -0.060 | 0.048 | 0.53797 | 2.08E-01 |
| rs7356980 | 6 | 26396398 | 3.16E-02 | 0.296 | 0.137 | 1.1 | 6.87E-01 | -0.056 | 0.139 | 2.3 | 0.122 | 0.098 | 0.07162 | 2.12E-01 |
| rs2066446 | 1 | 67781117 | 1.67E-01 | -0.052 | 0.037 | 18.4 | 8.77E-01 | -0.008 | 0.054 | 19.6 | -0.037 | 0.031 | 0.5076 | 2.21E-01 |
| rs12364363 | 11 | 119500880 | 3.64E-02 | -0.061 | 0.029 | 48.7 | 3.78E-01 | 0.037 | 0.042 | 47.1 | -0.029 | 0.024 | 0.05463 | 2.24E-01 |
| rs5756419 | 22 | 37346295 | 3.90E-01 | -0.027 | 0.031 | 31.7 | 3.72E-01 | -0.041 | 0.046 | 32.9 | -0.031 | 0.026 | 0.79763 | 2.25E-01 |
| rs4081918 | 19 | 45373739 | 8.22E-01 | -0.012 | 0.052 | 8.5 | 8.68E-02 | -0.120 | 0.070 | 9.2 | -0.050 | 0.041 | 0.21244 | 2.30E-01 |
| rs2226299 | 21 | 34698924 | 3.88E-01 | -0.031 | 0.036 | 17.8 | 3.93E-01 | -0.046 | 0.054 | 18.6 | -0.036 | 0.030 | 0.82033 | 2.32E-01 |
| rs8039462 | 15 | 89181415 | 3.85E-01 | -0.025 | 0.029 | 47.2 | 4.04E-01 | -0.034 | 0.041 | 46.5 | -0.028 | 0.024 | 0.85151 | 2.34E-01 |
| rs2229113 | 11 | 117869670 | 3.97E-01 | 0.026 | 0.030 | 32.1 | 3.85E-01 | 0.040 | 0.046 | 30.3 | 0.030 | 0.025 | 0.79599 | 2.35E-01 |
| rs4518487 | 6 | 26467531 | 2.15E-01 | 0.045 | 0.036 | 19.5 | 8.08E-01 | 0.013 | 0.055 | 18.5 | 0.035 | 0.030 | 0.63177 | 2.42E-01 |
| rs8104483 | 19 | 45372354 | 2.43E-01 | -0.038 | 0.033 | 25.5 | 7.06E-01 | -0.017 | 0.046 | 27.2 | -0.031 | 0.027 | 0.70917 | 2.42E-01 |
| rs416560 | 6 | 29648785 | 3.02E-01 | 0.113 | 0.109 | 1.7 | 5.67E-01 | 0.081 | 0.141 | 2.3 | 0.101 | 0.086 | 0.85938 | 2.43E-01 |
| rs11568820 | 12 | 48302545 | 7.06E-02 | 0.067 | 0.037 | 19.1 | 5.79E-01 | -0.029 | 0.053 | 20.3 | 0.035 | 0.030 | 0.13475 | 2.46E-01 |
| rs2350270 | 15 | 89171891 | 6.70E-01 | -0.013 | 0.031 | 29.0 | 5.97E-03 | 0.129 | 0.047 | 27.1 | 0.030 | 0.026 | 0.01103 | 2.46E-01 |
| rs17690122 | 5 | 131867835 | 5.44E-02 | 0.068 | 0.035 | 19.6 | 3.26E-01 | -0.059 | 0.060 | 15.6 | 0.035 | 0.030 | 0.06753 | 2.47E-01 |
| rs9461254 | 6 | 26469445 | 2.10E-03 | 0.476 | 0.154 | 0.9 | 3.93E-02 | -0.406 | 0.197 | 1.3 | 0.140 | 0.121 | 0.00041 | 2.51E-01 |
| rs9288918 | 3 | 110814079 | 1.97E-01 | 0.106 | 0.082 | 3.0 | 8.43E-01 | 0.021 | 0.108 | 4.1 | 0.075 | 0.066 | 0.53274 | 2.51E-01 |
| rs10774671 | 12 | 113357193 | 6.03E-01 | 0.016 | 0.031 | 35.4 | 2.13E-01 | 0.054 | 0.043 | 35.7 | 0.029 | 0.025 | 0.47419 | 2.52E-01 |
| rs5746051 | 1 | 12261972 | 5.02E-01 | 0.024 | 0.035 | 19.6 | 2.75E-01 | 0.063 | 0.057 | 17.6 | 0.034 | 0.030 | 0.56222 | 2.52E-01 |
| rs2660 | 12 | 113357442 | 6.35E-01 | 0.015 | 0.031 | 35.2 | 1.95E-01 | 0.056 | 0.043 | 35.3 | 0.029 | 0.025 | 0.43396 | 2.55E-01 |
| rs228937 | 22 | 37520971 | 3.51E-01 | 0.032 | 0.034 | 21.5 | 5.18E-01 | 0.033 | 0.051 | 22.5 | 0.032 | 0.028 | 0.98959 | 2.56E-01 |
| rs78201038 | 11 | 119499316 | 9.54E-01 | -0.002 | 0.043 | 11.8 | 2.77E-02 | 0.150 | 0.068 | 10.1 | 0.041 | 0.036 | 0.05789 | 2.58E-01 |
| rs2072803 | 6 | 26392515 | 6.61E-01 | 0.019 | 0.043 | 13.0 | 1.47E-01 | 0.104 | 0.072 | 9.8 | 0.042 | 0.037 | 0.31056 | 2.59E-01 |
| rs5746056 | 1 | 12262689 | 5.20E-01 | 0.020 | 0.031 | 27.7 | 2.77E-01 | 0.053 | 0.049 | 25.0 | 0.030 | 0.026 | 0.56739 | 2.59E-01 |
| rs2834209 | 21 | 34752794 | 5.96E-01 | -0.016 | 0.030 | 32.7 | 2.32E-01 | -0.053 | 0.045 | 33.5 | -0.028 | 0.025 | 0.49153 | 2.65E-01 |
| rs2243599 | 21 | 34723494 | 8.84E-01 | 0.004 | 0.029 | 41.2 | 2.99E-02 | -0.092 | 0.042 | 39.8 | -0.027 | 0.024 | 0.06053 | 2.65E-01 |
| rs1293762 | 12 | 113430836 | 2.30E-01 | 0.035 | 0.029 | 45.0 | 8.26E-01 | 0.009 | 0.042 | 44.0 | 0.027 | 0.024 | 0.61127 | 2.67E-01 |
| rs2201584 | 1 | 67787715 | 1.43E-01 | -0.053 | 0.037 | 17.2 | 7.98E-01 | 0.015 | 0.058 | 16.1 | -0.034 | 0.031 | 0.31993 | 2.69E-01 |
| rs735240 | 19 | 7813336 | 4.28E-01 | -0.023 | 0.029 | 43.6 | 4.25E-01 | -0.034 | 0.043 | 42.4 | -0.026 | 0.024 | 0.82296 | 2.70E-01 |
| rs2512143 | 11 | 117860582 | 3.75E-01 | 0.027 | 0.030 | 32.7 | 5.19E-01 | 0.029 | 0.045 | 31.4 | 0.028 | 0.025 | 0.96701 | 2.73E-01 |
| rs3130250 | 6 | 29625001 | 2.03E-01 | -0.048 | 0.037 | 17.1 | 9.51E-01 | -0.003 | 0.056 | 15.4 | -0.034 | 0.031 | 0.51303 | 2.74E-01 |
| rs72842972 | 6 | 26398786 | 3.57E-01 | -0.072 | 0.078 | 3.2 | 5.59E-01 | -0.056 | 0.096 | 5.2 | -0.066 | 0.061 | 0.90086 | 2.78E-01 |
| rs72500819 | 6 | 26467089 | 1.99E-01 | 0.443 | 0.345 | 0.2 | 9.60E-01 | 0.024 | 0.486 | 0.2 | 0.303 | 0.281 | 0.48224 | 2.82E-01 |
| rs12483293 | 21 | 34716789 | 1.36E-01 | -0.046 | 0.031 | 28.2 | 7.77E-01 | 0.013 | 0.046 | 30.0 | -0.028 | 0.026 | 0.2825 | 2.84E-01 |
| rs66757203 | 6 | 26454956 | 3.24E-01 | 0.045 | 0.046 | 11.5 | 6.57E-01 | 0.032 | 0.071 | 9.0 | 0.041 | 0.038 | 0.87448 | 2.84E-01 |
| rs12722596 | 10 | 6056294 | 1.48E-01 | -0.068 | 0.047 | 10.5 | 8.22E-01 | 0.016 | 0.069 | 11.2 | -0.042 | 0.039 | 0.31557 | 2.86E-01 |
| rs8134731 | 21 | 34671532 | 4.62E-01 | -0.026 | 0.036 | 18.2 | 4.23E-01 | -0.043 | 0.054 | 18.8 | -0.032 | 0.030 | 0.7949 | 2.91E-01 |
| rs412776 | 19 | 45379516 | 3.10E-01 | 0.047 | 0.046 | 10.6 | 7.25E-01 | 0.024 | 0.067 | 10.4 | 0.039 | 0.038 | 0.77684 | 2.99E-01 |
| rs1477848 | 3 | 110817982 | 6.42E-01 | 0.027 | 0.059 | 6.5 | 2.72E-01 | 0.088 | 0.080 | 7.7 | 0.049 | 0.047 | 0.54446 | 3.04E-01 |
| rs10469291 | 19 | 45156839 | 1.08E-01 | -0.174 | 0.108 | 1.5 | 7.07E-01 | 0.051 | 0.136 | 2.5 | -0.087 | 0.084 | 0.19477 | 3.06E-01 |
| rs9426830 | 1 | 154594690 | 4.94E-01 | -0.019 | 0.028 | 49.6 | 4.12E-01 | 0.034 | 0.042 | 46.1 | 0.024 | 0.023 | 0.76063 | 3.06E-01 |
| rs3181216 | 5 | 158752978 | 7.47E-01 | 0.010 | 0.031 | 30.1 | 1.82E-01 | 0.060 | 0.045 | 29.4 | 0.026 | 0.025 | 0.35618 | 3.08E-01 |
| rs17384360 | 1 | 161030806 | 9.36E-01 | 0.003 | 0.032 | 28.9 | 9.27E-02 | 0.078 | 0.047 | 25.8 | 0.027 | 0.026 | 0.17871 | 3.09E-01 |
| rs4803767 | 19 | 45372959 | 3.68E-01 | -0.030 | 0.033 | 24.4 | 6.33E-01 | -0.022 | 0.047 | 26.1 | -0.028 | 0.027 | 0.89242 | 3.12E-01 |
| rs12125166 | 1 | 154582129 | 5.00E-01 | -0.019 | 0.028 | 49.6 | 4.34E-01 | 0.033 | 0.042 | 45.8 | 0.023 | 0.023 | 0.78308 | 3.19E-01 |
| rs735239 | 19 | 7813268 | 5.50E-01 | -0.018 | 0.030 | 33.6 | 3.73E-01 | -0.040 | 0.045 | 35.0 | -0.025 | 0.025 | 0.68576 | 3.20E-01 |
| rs3829260 | 11 | 119598729 | 8.21E-01 | -0.006 | 0.028 | 44.8 | 1.53E-01 | -0.059 | 0.041 | 49.9 | -0.023 | 0.023 | 0.29108 | 3.21E-01 |
| rs8105572 | 19 | 7809327 | 2.44E-01 | -0.051 | 0.044 | 11.8 | 9.79E-01 | -0.002 | 0.065 | 12.5 | -0.036 | 0.036 | 0.53063 | 3.25E-01 |
| rs7433877 | 3 | 110797337 | 3.11E-01 | 0.094 | 0.093 | 2.5 | 7.67E-01 | 0.036 | 0.120 | 3.2 | 0.072 | 0.074 | 0.70005 | 3.25E-01 |
| rs2508450 | 11 | 117863829 | 5.92E-01 | 0.015 | 0.029 | 43.5 | 3.40E-01 | 0.040 | 0.042 | 43.0 | 0.023 | 0.024 | 0.62761 | 3.26E-01 |
| rs73006720 | 11 | 119609314 | 7.27E-01 | -0.010 | 0.029 | 39.5 | 2.21E-01 | -0.052 | 0.042 | 45.9 | -0.023 | 0.024 | 0.41647 | 3.27E-01 |
| rs1800890 | 1 | 206949365 | 9.62E-01 | -0.001 | 0.029 | 39.2 | 9.34E-02 | -0.073 | 0.044 | 36.2 | -0.023 | 0.024 | 0.17003 | 3.31E-01 |
| rs1614887 | 6 | 26393021 | 2.44E-01 | -0.033 | 0.028 | 46.0 | 9.86E-01 | -0.001 | 0.041 | 44.4 | -0.023 | 0.023 | 0.51758 | 3.32E-01 |
| rs2834176 | 21 | 34676370 | 9.15E-01 | 0.003 | 0.029 | 41.4 | 6.57E-02 | -0.077 | 0.042 | 40.8 | -0.023 | 0.024 | 0.1149 | 3.35E-01 |
| rs80214591 | 11 | 119542830 | 1.33E-01 | 0.893 | 0.594 | 0.1 | 7.78E-01 | -0.194 | 0.690 | 0.1 | 0.430 | 0.450 | 0.23182 | 3.39E-01 |
| rs75072970 | 1 | 161058351 | 8.32E-01 | 0.015 | 0.072 | 4.1 | 4.45E-02 | -0.217 | 0.108 | 4.1 | -0.056 | 0.060 | 0.07306 | 3.46E-01 |
| rs2834166 | 21 | 34626654 | 2.14E-01 | 0.038 | 0.031 | 34.2 | 9.06E-01 | -0.005 | 0.043 | 34.2 | 0.023 | 0.025 | 0.41291 | 3.46E-01 |
| rs7398676 | 12 | 53630558 | 2.67E-01 | 0.031 | 0.028 | 45.5 | 9.79E-01 | 0.001 | 0.042 | 48.0 | 0.022 | 0.023 | 0.55577 | 3.46E-01 |
| rs2857602 | 6 | 31533378 | 6.26E-01 | 0.015 | 0.030 | 40.6 | 3.48E-01 | 0.040 | 0.042 | 41.1 | 0.023 | 0.025 | 0.62782 | 3.47E-01 |
| rs9402877 | 6 | 137509075 | 2.92E-01 | -0.031 | 0.029 | 38.6 | 9.04E-01 | -0.005 | 0.043 | 38.2 | -0.023 | 0.024 | 0.62272 | 3.47E-01 |
| rs2384072 | 12 | 113367422 | 7.65E-01 | 0.009 | 0.030 | 36.3 | 2.32E-01 | 0.052 | 0.043 | 36.4 | 0.023 | 0.025 | 0.42032 | 3.50E-01 |
| rs10892429 | 11 | 119548052 | 1.74E-02 | 0.071 | 0.030 | 35.8 | 7.22E-02 | -0.078 | 0.043 | 35.1 | 0.023 | 0.025 | 0.00453 | 3.51E-01 |
| rs2535257 | 6 | 29629961 | 3.83E-01 | 0.106 | 0.121 | 1.4 | 6.79E-01 | 0.057 | 0.139 | 2.4 | 0.085 | 0.091 | 0.79158 | 3.53E-01 |
| rs459482 | 21 | 42793791 | 3.56E-01 | -0.026 | 0.029 | 44.5 | 7.74E-01 | -0.012 | 0.043 | 43.5 | -0.022 | 0.024 | 0.78775 | 3.53E-01 |
| rs7970314 | 12 | 48308174 | 5.97E-02 | 0.069 | 0.036 | 20.2 | 2.72E-01 | -0.058 | 0.053 | 21.4 | 0.028 | 0.030 | 0.04787 | 3.56E-01 |
| rs3136534 | 4 | 123369776 | 4.99E-01 | -0.021 | 0.031 | 34.9 | 5.21E-01 | -0.029 | 0.045 | 34.7 | -0.023 | 0.025 | 0.88454 | 3.57E-01 |
| rs7278931 | 21 | 34672447 | 4.86E-01 | -0.026 | 0.038 | 17.5 | 5.46E-01 | -0.033 | 0.054 | 18.5 | -0.028 | 0.031 | 0.91978 | 3.60E-01 |
| rs59832923 | 11 | 119501980 | 3.44E-01 | -0.566 | 0.598 | 0.1 | 4.83E-02 | 0.991 | 0.501 | 0.2 | 0.350 | 0.384 | 0.04594 | 3.63E-01 |
| rs7277852 | 21 | 34679203 | 7.04E-01 | 0.022 | 0.059 | 6.4 | 2.89E-01 | 0.092 | 0.086 | 6.3 | 0.044 | 0.048 | 0.50576 | 3.63E-01 |
| rs7252229 | 19 | 7812181 | 3.08E-01 | -0.044 | 0.043 | 12.2 | 9.19E-01 | -0.007 | 0.064 | 13.1 | -0.032 | 0.036 | 0.62812 | 3.66E-01 |
| rs478143 | 1 | 12245708 | 7.21E-01 | 0.011 | 0.032 | 26.7 | 2.72E-01 | 0.053 | 0.048 | 24.1 | 0.024 | 0.026 | 0.47086 | 3.66E-01 |
| rs12416901 | 11 | 119587095 | 2.14E-01 | 0.108 | 0.087 | 2.8 | 7.55E-01 | -0.045 | 0.144 | 2.2 | 0.067 | 0.075 | 0.36322 | 3.67E-01 |
| rs421812 | 19 | 45380545 | 4.74E-01 | 0.023 | 0.032 | 29.5 | 5.87E-01 | 0.025 | 0.046 | 28.4 | 0.024 | 0.026 | 0.96634 | 3.69E-01 |
| rs1547518 | 3 | 110794258 | 7.09E-01 | 0.020 | 0.054 | 7.6 | 3.09E-01 | 0.077 | 0.076 | 9.0 | 0.039 | 0.044 | 0.53957 | 3.71E-01 |
| rs6870828 | 5 | 158738512 | 6.58E-01 | -0.012 | 0.028 | 47.8 | 3.48E-01 | 0.039 | 0.041 | 48.7 | 0.021 | 0.023 | 0.59735 | 3.71E-01 |
| rs11576830 | 1 | 161068836 | 6.27E-01 | 0.015 | 0.031 | 30.0 | 3.76E-01 | 0.041 | 0.047 | 28.1 | 0.023 | 0.026 | 0.63657 | 3.72E-01 |
| rs29270 | 6 | 29617400 | 9.62E-01 | -0.003 | 0.066 | 4.9 | 1.29E-01 | 0.130 | 0.085 | 6.4 | 0.047 | 0.052 | 0.21755 | 3.72E-01 |
| rs5980742 | X | 70321631 | 5.28E-01 | 0.021 | 0.033 | 0.6 | 5.08E-01 | 0.036 | 0.054 | 0.7 | 0.025 | 0.028 | 0.80986 | 3.77E-01 |
| rs376319 | 6 | 29644633 | 2.03E-01 | 0.044 | 0.035 | 23.0 | 7.78E-01 | -0.014 | 0.049 | 24.5 | 0.025 | 0.028 | 0.33494 | 3.79E-01 |
| rs9882945 | 3 | 110839660 | 3.14E-01 | 0.084 | 0.084 | 3.0 | 9.05E-01 | 0.013 | 0.113 | 3.7 | 0.059 | 0.067 | 0.61355 | 3.79E-01 |
| rs10892440 | 11 | 119584274 | 2.66E-01 | 0.097 | 0.087 | 2.8 | 9.06E-01 | -0.016 | 0.139 | 2.4 | 0.065 | 0.074 | 0.48917 | 3.79E-01 |
| rs17526942 | 3 | 25617403 | 6.32E-01 | 0.022 | 0.045 | 11.7 | 3.92E-01 | 0.057 | 0.067 | 11.3 | 0.033 | 0.037 | 0.65795 | 3.81E-01 |
| rs9358944 | 6 | 26469875 | 6.16E-01 | 0.019 | 0.037 | 18.5 | 4.08E-01 | 0.046 | 0.056 | 17.0 | 0.027 | 0.031 | 0.6801 | 3.81E-01 |
| rs7356988 | 6 | 26396727 | 4.71E-01 | 0.021 | 0.029 | 40.8 | 6.24E-01 | 0.021 | 0.043 | 42.5 | 0.021 | 0.024 | 0.99794 | 3.83E-01 |
| rs469483 | 21 | 42818515 | 3.83E-01 | 0.026 | 0.029 | 40.9 | 7.96E-01 | 0.011 | 0.044 | 41.7 | 0.021 | 0.024 | 0.78994 | 3.84E-01 |
| rs1351049 | 3 | 110842276 | 7.87E-01 | 0.016 | 0.058 | 6.7 | 2.72E-01 | 0.088 | 0.080 | 7.7 | 0.041 | 0.047 | 0.46546 | 3.86E-01 |
| rs2243148 | 3 | 159715411 | 1.12E-01 | 0.048 | 0.030 | 29.9 | 3.61E-01 | -0.044 | 0.048 | 28.6 | 0.022 | 0.025 | 0.10491 | 3.90E-01 |
| rs34953153 | 11 | 119530129 | 2.30E-01 | 0.061 | 0.051 | 8.7 | 7.96E-01 | -0.020 | 0.077 | 8.8 | 0.036 | 0.043 | 0.37834 | 3.91E-01 |
| rs9467749 | 6 | 26395918 | 7.26E-02 | 0.269 | 0.150 | 1.0 | 5.00E-01 | -0.109 | 0.161 | 1.7 | 0.094 | 0.110 | 0.08567 | 3.92E-01 |
| rs76715864 | 6 | 26463416 | 6.97E-01 | -0.023 | 0.058 | 6.8 | 3.51E-01 | -0.078 | 0.083 | 6.9 | -0.041 | 0.048 | 0.58721 | 3.94E-01 |
| rs3810143 | 19 | 45349402 | 8.99E-01 | 0.004 | 0.029 | 42.1 | 1.91E-01 | 0.055 | 0.042 | 41.6 | 0.020 | 0.024 | 0.31461 | 3.96E-01 |
| rs10403018 | 19 | 7807550 | 9.76E-01 | -0.007 | 0.244 | 0.3 | 1.21E-01 | 0.557 | 0.358 | 0.4 | 0.171 | 0.201 | 0.19269 | 3.96E-01 |
| rs11881682 | 19 | 7815376 | 1.87E-01 | -0.052 | 0.039 | 14.9 | 6.48E-01 | 0.027 | 0.059 | 15.1 | -0.028 | 0.033 | 0.26689 | 3.96E-01 |
| rs28615360 | 19 | 45344722 | 9.61E-01 | 0.007 | 0.134 | 1.1 | 1.52E-01 | -0.244 | 0.170 | 1.6 | -0.089 | 0.105 | 0.24736 | 3.97E-01 |
| rs78736195 | 1 | 161067637 | 3.39E-01 | 0.059 | 0.061 | 5.8 | 9.39E-01 | 0.007 | 0.093 | 5.6 | 0.043 | 0.051 | 0.6429 | 4.01E-01 |
| rs2291842 | 11 | 5719667 | 3.08E-01 | 0.038 | 0.037 | 17.1 | 9.75E-01 | 0.002 | 0.050 | 22.4 | 0.025 | 0.030 | 0.56006 | 4.02E-01 |
| rs4619159 | 11 | 119514953 | 4.89E-01 | -0.171 | 0.247 | 0.3 | 6.32E-01 | -0.136 | 0.283 | 0.6 | -0.156 | 0.186 | 0.92448 | 4.03E-01 |
| rs1900173 | 5 | 55240006 | 5.17E-01 | 0.036 | 0.056 | 7.1 | 5.94E-01 | 0.044 | 0.082 | 7.2 | 0.038 | 0.046 | 0.93884 | 4.03E-01 |
| rs11672399 | 19 | 45377098 | 9.45E-01 | 0.004 | 0.055 | 7.4 | 1.32E-01 | -0.114 | 0.076 | 7.9 | -0.037 | 0.045 | 0.20697 | 4.05E-01 |
| rs9616 | 1 | 154555733 | 3.18E-01 | -0.031 | 0.031 | 28.3 | 9.92E-01 | 0.000 | 0.046 | 28.6 | -0.021 | 0.026 | 0.57978 | 4.06E-01 |
| rs471692 | 17 | 38556770 | 3.30E-01 | 0.035 | 0.036 | 20.2 | 9.74E-01 | 0.002 | 0.054 | 19.1 | 0.025 | 0.030 | 0.61005 | 4.06E-01 |
| rs1293755 | 12 | 113435450 | 3.45E-01 | 0.029 | 0.031 | 30.9 | 9.39E-01 | 0.003 | 0.045 | 31.6 | 0.021 | 0.025 | 0.63865 | 4.11E-01 |
| rs7952366 | 11 | 119597943 | 8.75E-01 | -0.005 | 0.029 | 39.6 | 2.22E-01 | -0.052 | 0.042 | 45.9 | -0.020 | 0.024 | 0.35714 | 4.13E-01 |
| rs11465413 | 19 | 7805951 | 4.06E-01 | 0.040 | 0.048 | 9.2 | 8.61E-01 | 0.016 | 0.091 | 5.7 | 0.035 | 0.042 | 0.81721 | 4.14E-01 |
| rs11217397 | 11 | 119556585 | 6.75E-01 | -0.176 | 0.420 | 0.1 | 4.74E-01 | -0.286 | 0.399 | 0.3 | -0.234 | 0.289 | 0.84961 | 4.18E-01 |
| rs73575004 | 11 | 119558829 | 6.80E-01 | -0.174 | 0.421 | 0.1 | 4.74E-01 | -0.286 | 0.399 | 0.3 | -0.233 | 0.290 | 0.84635 | 4.21E-01 |
| rs12418662 | 11 | 119596772 | 8.75E-01 | -0.005 | 0.029 | 39.6 | 2.37E-01 | -0.050 | 0.042 | 45.9 | -0.019 | 0.024 | 0.37532 | 4.24E-01 |
| rs9358945 | 6 | 26472114 | 6.82E-01 | 0.015 | 0.037 | 18.4 | 4.08E-01 | 0.046 | 0.056 | 17.0 | 0.025 | 0.031 | 0.64133 | 4.25E-01 |
| rs2009658 | 6 | 31538244 | 8.34E-01 | 0.008 | 0.040 | 15.5 | 2.55E-01 | 0.069 | 0.061 | 14.9 | 0.026 | 0.033 | 0.40039 | 4.25E-01 |
| rs11217430 | 11 | 119595209 | 9.09E-01 | -0.003 | 0.029 | 39.5 | 2.15E-01 | -0.052 | 0.042 | 45.8 | -0.019 | 0.024 | 0.33753 | 4.26E-01 |
| rs2116703 | 3 | 25542571 | 9.95E-01 | 0.000 | 0.037 | 17.0 | 1.64E-01 | -0.077 | 0.055 | 19.5 | -0.024 | 0.031 | 0.24932 | 4.32E-01 |
| rs56668317 | 3 | 110842878 | 8.50E-01 | 0.010 | 0.055 | 7.3 | 2.83E-01 | 0.082 | 0.077 | 8.6 | 0.035 | 0.045 | 0.44705 | 4.33E-01 |
| rs76366838 | 19 | 45399896 | 5.88E-01 | -0.047 | 0.087 | 2.8 | 5.62E-01 | -0.066 | 0.115 | 3.4 | -0.054 | 0.069 | 0.89421 | 4.34E-01 |
| rs9393725 | 6 | 26485779 | 8.03E-01 | -0.028 | 0.114 | 1.6 | 7.07E-02 | 0.318 | 0.175 | 1.5 | 0.074 | 0.096 | 0.09793 | 4.37E-01 |
| rs78157313 | 3 | 110836355 | 4.13E-01 | 0.141 | 0.173 | 0.6 | 9.06E-01 | 0.036 | 0.310 | 0.5 | 0.116 | 0.151 | 0.76751 | 4.40E-01 |
| rs75054607 | 3 | 110792422 | 4.14E-01 | 0.141 | 0.173 | 0.6 | 9.06E-01 | 0.036 | 0.310 | 0.5 | 0.116 | 0.151 | 0.7683 | 4.41E-01 |
| rs76464851 | 3 | 110845612 | 4.14E-01 | 0.141 | 0.173 | 0.6 | 9.06E-01 | 0.036 | 0.310 | 0.5 | 0.116 | 0.151 | 0.7683 | 4.41E-01 |
| rs13195402 | 6 | 26463575 | 3.30E-01 | 0.047 | 0.048 | 10.2 | 9.10E-01 | -0.009 | 0.077 | 7.8 | 0.031 | 0.041 | 0.54187 | 4.42E-01 |
| rs17860160 | 21 | 34611320 | 4.28E-01 | -0.029 | 0.037 | 16.7 | 8.45E-01 | -0.011 | 0.055 | 18.2 | -0.024 | 0.031 | 0.78383 | 4.42E-01 |
| rs4936496 | 11 | 119601313 | 9.99E-01 | 0.000 | 0.029 | 42.5 | 1.77E-01 | -0.057 | 0.042 | 48.1 | -0.018 | 0.024 | 0.26459 | 4.44E-01 |
| rs4409845 | 11 | 119582832 | 2.71E-01 | 0.096 | 0.087 | 2.8 | 7.41E-01 | -0.047 | 0.142 | 2.3 | 0.057 | 0.074 | 0.39058 | 4.44E-01 |
| rs1796519 | 6 | 26404680 | 2.40E-01 | 0.048 | 0.041 | 14.1 | 7.20E-01 | -0.021 | 0.059 | 14.2 | 0.026 | 0.034 | 0.33545 | 4.44E-01 |
| rs4938718 | 11 | 119601497 | 9.99E-01 | 0.000 | 0.029 | 42.5 | 1.77E-01 | -0.057 | 0.042 | 48.1 | -0.018 | 0.024 | 0.26421 | 4.45E-01 |
| rs6800657 | 3 | 159699372 | 1.46E-01 | 0.068 | 0.047 | 10.5 | 4.85E-01 | -0.044 | 0.064 | 11.7 | 0.029 | 0.038 | 0.15473 | 4.47E-01 |
| rs1800630 | 6 | 31542476 | 8.47E-01 | 0.008 | 0.040 | 15.5 | 2.77E-01 | 0.066 | 0.061 | 14.7 | 0.025 | 0.033 | 0.41963 | 4.49E-01 |
| rs10892428 | 11 | 119536932 | 6.80E-01 | -0.174 | 0.421 | 0.1 | 4.98E-01 | -0.331 | 0.488 | 0.2 | -0.241 | 0.319 | 0.80763 | 4.50E-01 |
| rs11217385 | 11 | 119539548 | 6.80E-01 | -0.174 | 0.421 | 0.1 | 4.98E-01 | -0.331 | 0.488 | 0.2 | -0.241 | 0.319 | 0.80763 | 4.50E-01 |
| rs11603655 | 11 | 119534730 | 6.80E-01 | -0.174 | 0.421 | 0.1 | 4.98E-01 | -0.331 | 0.488 | 0.2 | -0.241 | 0.319 | 0.80763 | 4.50E-01 |
| rs11604525 | 11 | 119524090 | 6.80E-01 | -0.174 | 0.421 | 0.1 | 4.98E-01 | -0.331 | 0.488 | 0.2 | -0.241 | 0.319 | 0.80763 | 4.50E-01 |
| rs57959285 | 11 | 119521116 | 6.80E-01 | -0.174 | 0.421 | 0.1 | 4.98E-01 | -0.331 | 0.488 | 0.2 | -0.241 | 0.319 | 0.80763 | 4.50E-01 |
| rs60008550 | 11 | 119530672 | 6.80E-01 | -0.174 | 0.421 | 0.1 | 4.98E-01 | -0.331 | 0.488 | 0.2 | -0.241 | 0.319 | 0.80763 | 4.50E-01 |
| rs73571285 | 11 | 119515987 | 6.80E-01 | -0.174 | 0.421 | 0.1 | 4.98E-01 | -0.331 | 0.488 | 0.2 | -0.241 | 0.319 | 0.80763 | 4.50E-01 |
| rs73571286 | 11 | 119516222 | 6.80E-01 | -0.174 | 0.421 | 0.1 | 4.98E-01 | -0.331 | 0.488 | 0.2 | -0.241 | 0.319 | 0.80763 | 4.50E-01 |
| rs73571287 | 11 | 119516489 | 6.80E-01 | -0.174 | 0.421 | 0.1 | 4.98E-01 | -0.331 | 0.488 | 0.2 | -0.241 | 0.319 | 0.80763 | 4.50E-01 |
| rs73573188 | 11 | 119534066 | 6.80E-01 | -0.174 | 0.421 | 0.1 | 4.98E-01 | -0.331 | 0.488 | 0.2 | -0.241 | 0.319 | 0.80763 | 4.50E-01 |
| rs7943014 | 11 | 119529275 | 6.80E-01 | -0.174 | 0.421 | 0.1 | 4.98E-01 | -0.331 | 0.488 | 0.2 | -0.241 | 0.319 | 0.80763 | 4.50E-01 |
| rs7944707 | 11 | 119532600 | 6.80E-01 | -0.174 | 0.421 | 0.1 | 4.98E-01 | -0.331 | 0.488 | 0.2 | -0.241 | 0.319 | 0.80763 | 4.50E-01 |
| rs7944565 | 11 | 119511611 | 6.80E-01 | -0.173 | 0.421 | 0.1 | 4.98E-01 | -0.331 | 0.488 | 0.2 | -0.240 | 0.319 | 0.80716 | 4.51E-01 |
| rs12006123 | 9 | 32456017 | 1.72E-01 | -0.047 | 0.034 | 21.4 | 4.36E-01 | 0.043 | 0.056 | 19.8 | -0.022 | 0.029 | 0.1676 | 4.51E-01 |
| rs7931981 | 11 | 119515398 | 6.84E-01 | -0.171 | 0.421 | 0.1 | 4.98E-01 | -0.331 | 0.488 | 0.2 | -0.239 | 0.319 | 0.80468 | 4.53E-01 |
| rs29235 | 6 | 29624078 | 6.42E-01 | 0.035 | 0.075 | 4.0 | 5.15E-01 | 0.073 | 0.112 | 3.8 | 0.047 | 0.063 | 0.77896 | 4.54E-01 |
| rs72935989 | 3 | 110800763 | 3.99E-01 | 0.081 | 0.096 | 2.3 | 8.77E-01 | 0.018 | 0.115 | 3.6 | 0.055 | 0.074 | 0.67305 | 4.55E-01 |
| rs35879138 | 19 | 45383139 | 2.46E-01 | 0.053 | 0.045 | 10.8 | 6.81E-01 | -0.028 | 0.068 | 10.1 | 0.028 | 0.038 | 0.32437 | 4.59E-01 |
| rs60900866 | 6 | 26455425 | 5.27E-03 | 0.483 | 0.173 | 0.7 | 3.93E-02 | -0.406 | 0.197 | 1.3 | 0.096 | 0.130 | 0.00068 | 4.60E-01 |
| rs41266839 | 6 | 26409890 | 4.10E-01 | 0.040 | 0.048 | 10.1 | 9.78E-01 | 0.002 | 0.077 | 7.9 | 0.029 | 0.041 | 0.67913 | 4.75E-01 |
| rs72935984 | 3 | 110798886 | 4.83E-01 | 0.213 | 0.303 | 0.2 | 7.82E-01 | 0.097 | 0.349 | 0.4 | 0.163 | 0.229 | 0.80162 | 4.77E-01 |
| rs590977 | 1 | 12255360 | 6.69E-01 | 0.015 | 0.036 | 19.2 | 5.20E-01 | 0.036 | 0.055 | 17.7 | 0.021 | 0.030 | 0.75989 | 4.78E-01 |
| rs474247 | 1 | 12246175 | 7.92E-01 | 0.009 | 0.036 | 19.5 | 1.17E-01 | -0.078 | 0.050 | 23.1 | -0.021 | 0.029 | 0.15354 | 4.79E-01 |
| rs677844 | 1 | 12244318 | 9.95E-01 | 0.000 | 0.032 | 25.8 | 2.08E-01 | 0.061 | 0.048 | 23.1 | 0.019 | 0.027 | 0.29643 | 4.81E-01 |
| rs2834160 | 21 | 34620113 | 4.52E-01 | -0.027 | 0.036 | 17.0 | 9.00E-01 | -0.007 | 0.055 | 18.5 | -0.021 | 0.030 | 0.7546 | 4.87E-01 |
| rs283813 | 19 | 45389174 | 6.73E-01 | 0.022 | 0.052 | 8.3 | 5.23E-01 | 0.054 | 0.084 | 6.8 | 0.031 | 0.044 | 0.74716 | 4.87E-01 |
| rs13126816 | 4 | 186994178 | 5.13E-01 | -0.022 | 0.033 | 24.0 | 7.88E-01 | -0.013 | 0.050 | 25.2 | -0.019 | 0.028 | 0.89123 | 4.88E-01 |
| rs1552902 | 1 | 154608823 | 2.84E-01 | -0.032 | 0.029 | 33.0 | 7.34E-01 | 0.015 | 0.043 | 35.7 | -0.017 | 0.024 | 0.3738 | 4.91E-01 |
| rs73936968 | 19 | 45395816 | 2.74E-01 | -0.119 | 0.109 | 1.7 | 7.68E-01 | 0.041 | 0.139 | 2.4 | -0.058 | 0.086 | 0.36409 | 4.97E-01 |
| rs12626735 | 21 | 34815979 | 9.16E-01 | -0.004 | 0.034 | 23.2 | 2.88E-01 | -0.054 | 0.051 | 22.1 | -0.019 | 0.028 | 0.40658 | 5.00E-01 |
| rs12116949 | 1 | 161042063 | 4.98E-01 | -0.022 | 0.033 | 28.8 | 8.39E-01 | -0.010 | 0.047 | 28.8 | -0.018 | 0.027 | 0.82876 | 5.01E-01 |
| rs897751 | 19 | 18193349 | 1.65E-01 | -0.039 | 0.028 | 44.6 | 3.66E-01 | 0.039 | 0.043 | 43.0 | -0.016 | 0.024 | 0.12935 | 5.03E-01 |
| rs3764874 | 12 | 6458274 | 2.89E-01 | 0.035 | 0.033 | 23.2 | 6.70E-01 | -0.022 | 0.051 | 20.4 | 0.018 | 0.027 | 0.35232 | 5.05E-01 |
| rs370705 | 19 | 45379638 | 6.71E-01 | 0.013 | 0.032 | 29.5 | 5.78E-01 | 0.026 | 0.046 | 28.5 | 0.017 | 0.026 | 0.82597 | 5.06E-01 |
| rs4802240 | 19 | 45352804 | 6.79E-01 | -0.012 | 0.029 | 41.6 | 5.72E-01 | -0.024 | 0.042 | 40.6 | -0.016 | 0.024 | 0.81592 | 5.08E-01 |
| rs1876795 | 11 | 119596147 | 9.28E-01 | 0.003 | 0.029 | 42.5 | 1.95E-01 | -0.054 | 0.042 | 48.2 | -0.016 | 0.024 | 0.26182 | 5.09E-01 |
| rs73572039 | 19 | 45342630 | 8.25E-01 | 0.010 | 0.046 | 10.7 | 4.06E-01 | 0.055 | 0.066 | 12.3 | 0.025 | 0.038 | 0.57945 | 5.10E-01 |
| rs35385129 | 19 | 45162189 | 4.97E-01 | 0.027 | 0.040 | 15.8 | 8.74E-01 | 0.009 | 0.058 | 15.8 | 0.021 | 0.033 | 0.79892 | 5.16E-01 |
| rs6456723 | 6 | 26455505 | 4.74E-01 | 0.027 | 0.037 | 17.6 | 9.11E-01 | 0.006 | 0.052 | 22.1 | 0.020 | 0.030 | 0.74334 | 5.17E-01 |
| rs7750281 | 6 | 26459015 | 5.52E-03 | 0.430 | 0.155 | 0.9 | 1.81E-02 | -0.447 | 0.188 | 1.4 | 0.077 | 0.120 | 0.00032 | 5.17E-01 |
| rs78757430 | 11 | 119505431 | 2.01E-01 | 0.763 | 0.595 | 0.1 | 6.17E-01 | -0.346 | 0.692 | 0.1 | 0.291 | 0.451 | 0.22468 | 5.19E-01 |
| rs17037696 | 1 | 12243274 | 9.68E-01 | -0.001 | 0.033 | 26.9 | 2.30E-01 | 0.058 | 0.048 | 23.0 | 0.017 | 0.027 | 0.30923 | 5.22E-01 |
| rs1799964 | 6 | 31542308 | 1.63E-01 | -0.049 | 0.035 | 19.9 | 3.37E-01 | 0.052 | 0.054 | 19.6 | -0.019 | 0.030 | 0.1169 | 5.22E-01 |
| rs4938713 | 11 | 119568994 | 4.29E-01 | -0.032 | 0.040 | 15.8 | 9.87E-01 | 0.001 | 0.058 | 14.5 | -0.021 | 0.033 | 0.64071 | 5.23E-01 |
| rs11217426 | 11 | 119590878 | 9.64E-01 | -0.001 | 0.029 | 39.7 | 2.92E-01 | -0.044 | 0.042 | 45.7 | -0.015 | 0.024 | 0.39891 | 5.24E-01 |
| rs2248412 | 21 | 34605531 | 3.80E-01 | 0.034 | 0.039 | 16.0 | 9.00E-01 | -0.007 | 0.053 | 17.7 | 0.020 | 0.031 | 0.53446 | 5.26E-01 |
| rs7624894 | 3 | 25642157 | 3.04E-01 | 0.050 | 0.049 | 9.8 | 7.34E-01 | -0.023 | 0.067 | 11.2 | 0.025 | 0.040 | 0.37858 | 5.29E-01 |
| rs60657140 | 11 | 119505336 | 8.37E-01 | -0.009 | 0.044 | 11.7 | 4.17E-01 | -0.054 | 0.066 | 11.2 | -0.023 | 0.036 | 0.57292 | 5.35E-01 |
| rs1058402 | 19 | 45150614 | 9.12E-01 | -0.008 | 0.068 | 4.5 | 2.24E-01 | 0.116 | 0.095 | 5.5 | 0.034 | 0.055 | 0.29173 | 5.35E-01 |
| rs2272020 | 19 | 45150433 | 9.12E-01 | -0.008 | 0.068 | 4.5 | 2.24E-01 | 0.116 | 0.095 | 5.5 | 0.034 | 0.055 | 0.29173 | 5.35E-01 |
| rs60052241 | 19 | 45151280 | 9.12E-01 | -0.008 | 0.068 | 4.5 | 2.24E-01 | 0.116 | 0.095 | 5.5 | 0.034 | 0.055 | 0.29173 | 5.35E-01 |
| rs2965157 | 19 | 45176340 | 4.05E-01 | 0.079 | 0.095 | 2.3 | 9.70E-01 | -0.004 | 0.115 | 3.6 | 0.045 | 0.073 | 0.57659 | 5.36E-01 |
| rs7280479 | 21 | 34611197 | 8.65E-01 | -0.008 | 0.047 | 10.9 | 1.88E-01 | 0.088 | 0.067 | 11.9 | 0.024 | 0.039 | 0.24006 | 5.36E-01 |
| rs6900118 | 6 | 26462641 | 6.53E-01 | 0.029 | 0.064 | 5.3 | 6.78E-01 | 0.038 | 0.091 | 6.0 | 0.032 | 0.053 | 0.93516 | 5.44E-01 |
| rs11217372 | 11 | 119519217 | 2.83E-01 | 0.055 | 0.051 | 8.4 | 6.64E-01 | -0.030 | 0.070 | 10.5 | 0.025 | 0.041 | 0.32282 | 5.44E-01 |
| rs2464288 | 12 | 113459379 | 6.38E-01 | 0.016 | 0.033 | 24.7 | 7.00E-01 | 0.019 | 0.050 | 23.8 | 0.017 | 0.028 | 0.95284 | 5.45E-01 |
| rs20337 | 11 | 119502870 | 8.34E-01 | -0.009 | 0.044 | 11.7 | 4.43E-01 | -0.050 | 0.066 | 11.4 | -0.022 | 0.036 | 0.60163 | 5.49E-01 |
| rs12788928 | 11 | 119514810 | 4.72E-01 | 0.038 | 0.053 | 8.1 | 9.95E-01 | 0.000 | 0.078 | 8.0 | 0.026 | 0.044 | 0.68925 | 5.50E-01 |
| rs2535250 | 6 | 29634417 | 6.53E-01 | 0.042 | 0.093 | 2.5 | 6.94E-01 | 0.045 | 0.114 | 3.4 | 0.043 | 0.072 | 0.98347 | 5.50E-01 |
| rs2857767 | 6 | 29634268 | 6.55E-01 | 0.042 | 0.093 | 2.5 | 6.94E-01 | 0.045 | 0.114 | 3.4 | 0.043 | 0.072 | 0.98205 | 5.51E-01 |
| rs3129063 | 6 | 29645613 | 3.16E-01 | 0.036 | 0.035 | 21.2 | 6.87E-01 | -0.021 | 0.051 | 22.1 | 0.017 | 0.029 | 0.36597 | 5.52E-01 |
| rs29231 | 6 | 29618525 | 9.03E-01 | 0.005 | 0.037 | 17.3 | 3.82E-01 | 0.048 | 0.055 | 18.5 | 0.018 | 0.031 | 0.5138 | 5.52E-01 |
| rs73573185 | 11 | 119531171 | 2.56E-01 | 0.339 | 0.298 | 0.2 | 6.74E-01 | -0.146 | 0.348 | 0.4 | 0.134 | 0.227 | 0.28929 | 5.55E-01 |
| rs7255063 | 19 | 45352419 | 7.22E-01 | -0.010 | 0.029 | 41.6 | 6.03E-01 | -0.022 | 0.042 | 40.7 | -0.014 | 0.024 | 0.82092 | 5.57E-01 |
| rs4233364 | 1 | 161054223 | 3.20E-01 | 0.045 | 0.046 | 11.1 | 6.36E-01 | -0.034 | 0.072 | 10.6 | 0.023 | 0.039 | 0.35081 | 5.57E-01 |
| rs10892438 | 11 | 119579346 | 6.54E-01 | -0.012 | 0.028 | 45.1 | 7.96E-02 | 0.074 | 0.042 | 39.8 | 0.013 | 0.023 | 0.08609 | 5.60E-01 |
| rs1001611 | 19 | 45346768 | 7.27E-01 | -0.010 | 0.029 | 41.4 | 6.03E-01 | -0.022 | 0.042 | 40.7 | -0.014 | 0.024 | 0.81734 | 5.60E-01 |
| rs80098994 | 6 | 26471471 | 1.75E-01 | -0.361 | 0.266 | 0.3 | 8.97E-02 | 0.328 | 0.193 | 1.0 | 0.091 | 0.156 | 0.03604 | 5.61E-01 |
| rs29269 | 6 | 29617747 | 9.56E-01 | 0.002 | 0.038 | 17.1 | 3.43E-01 | 0.052 | 0.055 | 17.8 | 0.018 | 0.031 | 0.45224 | 5.61E-01 |
| rs4804805 | 19 | 7816546 | 7.05E-01 | -0.012 | 0.032 | 30.8 | 6.41E-01 | -0.021 | 0.046 | 30.5 | -0.015 | 0.026 | 0.86463 | 5.65E-01 |
| rs7104562 | 11 | 119577324 | 3.38E-01 | 0.088 | 0.092 | 2.5 | 6.75E-01 | -0.059 | 0.141 | 2.3 | 0.044 | 0.077 | 0.38205 | 5.65E-01 |
| rs1871026 | 3 | 110783005 | 6.65E-01 | 0.027 | 0.062 | 5.5 | 7.01E-01 | 0.035 | 0.090 | 6.2 | 0.029 | 0.051 | 0.94307 | 5.66E-01 |
| rs1477844 | 3 | 110829831 | 9.94E-01 | 0.000 | 0.039 | 17.1 | 3.25E-01 | 0.054 | 0.055 | 19.9 | 0.018 | 0.032 | 0.42184 | 5.67E-01 |
| rs17503646 | 1 | 182539880 | 5.88E-01 | 0.023 | 0.042 | 13.5 | 7.85E-02 | -0.105 | 0.060 | 14.6 | -0.020 | 0.034 | 0.07941 | 5.69E-01 |
| rs404733 | 19 | 18169997 | 6.81E-01 | 0.011 | 0.027 | 49.2 | 6.81E-01 | 0.017 | 0.042 | 46.0 | 0.013 | 0.023 | 0.90464 | 5.69E-01 |
| rs12786505 | 11 | 119607659 | 9.98E-01 | 0.000 | 0.029 | 39.1 | 3.22E-01 | -0.042 | 0.042 | 45.8 | -0.013 | 0.024 | 0.41363 | 5.74E-01 |
| rs4445648 | 11 | 119529524 | 5.45E-01 | -0.022 | 0.036 | 21.6 | 9.14E-01 | -0.006 | 0.053 | 21.4 | -0.017 | 0.030 | 0.80014 | 5.75E-01 |
| rs4682233 | 3 | 110845394 | 5.90E-01 | 0.226 | 0.420 | 0.1 | 7.82E-01 | 0.097 | 0.349 | 0.4 | 0.150 | 0.268 | 0.81247 | 5.77E-01 |
| rs72937914 | 3 | 110839459 | 5.90E-01 | 0.226 | 0.420 | 0.1 | 7.82E-01 | 0.097 | 0.349 | 0.4 | 0.150 | 0.268 | 0.81247 | 5.77E-01 |
| rs395908 | 19 | 45373565 | 4.67E-01 | 0.029 | 0.040 | 15.3 | 9.53E-01 | -0.003 | 0.055 | 16.1 | 0.018 | 0.032 | 0.63621 | 5.78E-01 |
| rs394221 | 19 | 45368424 | 8.80E-01 | -0.004 | 0.028 | 41.4 | 4.50E-01 | -0.031 | 0.041 | 44.0 | -0.013 | 0.023 | 0.59212 | 5.79E-01 |
| rs10120977 | 9 | 21384363 | 4.53E-01 | 0.027 | 0.036 | 21.6 | 8.98E-01 | -0.007 | 0.053 | 20.5 | 0.016 | 0.030 | 0.60022 | 5.80E-01 |
| rs11600941 | 11 | 119582662 | 2.60E-01 | -0.388 | 0.344 | 0.2 | 5.21E-01 | 0.313 | 0.487 | 0.2 | -0.155 | 0.281 | 0.24018 | 5.82E-01 |
| rs12361192 | 11 | 119603642 | 9.20E-01 | 0.003 | 0.029 | 37.2 | 2.72E-01 | -0.046 | 0.042 | 43.4 | -0.013 | 0.024 | 0.33693 | 5.85E-01 |
| rs35182466 | 19 | 45341282 | 7.71E-01 | -0.008 | 0.029 | 39.9 | 5.93E-01 | -0.022 | 0.042 | 39.7 | -0.013 | 0.024 | 0.78292 | 5.87E-01 |
| rs3117286 | 6 | 29629774 | 7.05E-01 | 0.036 | 0.094 | 2.4 | 6.94E-01 | 0.045 | 0.114 | 3.4 | 0.039 | 0.073 | 0.95026 | 5.87E-01 |
| rs246841 | 5 | 131402186 | 7.44E-01 | -0.015 | 0.045 | 10.5 | 6.24E-01 | -0.035 | 0.071 | 9.7 | -0.021 | 0.038 | 0.81221 | 5.90E-01 |
| rs4938716 | 11 | 119576555 | 9.37E-01 | -0.002 | 0.029 | 35.0 | 2.67E-01 | 0.051 | 0.045 | 32.5 | 0.013 | 0.025 | 0.32819 | 5.92E-01 |
| rs4936490 | 11 | 119506749 | 9.88E-01 | -0.001 | 0.044 | 11.7 | 3.46E-01 | -0.063 | 0.066 | 10.9 | -0.019 | 0.036 | 0.43499 | 5.96E-01 |
| rs12815666 | 12 | 113415754 | 7.33E-01 | 0.014 | 0.041 | 13.8 | 6.54E-01 | 0.029 | 0.065 | 12.4 | 0.018 | 0.035 | 0.84558 | 5.97E-01 |
| rs1732778 | 12 | 113456925 | 6.18E-01 | 0.017 | 0.033 | 24.6 | 8.41E-01 | 0.010 | 0.050 | 23.8 | 0.015 | 0.028 | 0.91229 | 5.99E-01 |
| rs73004983 | 11 | 119572124 | 5.91E-01 | 0.031 | 0.057 | 6.7 | 8.73E-01 | 0.012 | 0.074 | 8.1 | 0.024 | 0.045 | 0.83993 | 6.01E-01 |
| rs60948164 | 19 | 45159936 | 8.78E-01 | -0.010 | 0.067 | 4.6 | 2.69E-01 | 0.103 | 0.093 | 5.8 | 0.028 | 0.054 | 0.32283 | 6.02E-01 |
| rs2089646 | 11 | 119512115 | 9.78E-01 | 0.001 | 0.051 | 8.3 | 3.28E-01 | -0.077 | 0.078 | 8.0 | -0.022 | 0.043 | 0.40306 | 6.09E-01 |
| rs416041 | 19 | 45370854 | 6.14E-01 | 0.015 | 0.029 | 40.6 | 8.69E-01 | 0.007 | 0.043 | 38.7 | 0.012 | 0.024 | 0.88702 | 6.09E-01 |
| rs35739046 | 19 | 45160523 | 6.31E-01 | 0.019 | 0.040 | 15.6 | 8.44E-01 | 0.011 | 0.058 | 16.0 | 0.017 | 0.033 | 0.91262 | 6.12E-01 |
| rs4938708 | 11 | 119560847 | 8.10E-01 | -0.007 | 0.029 | 39.1 | 1.90E-01 | 0.058 | 0.044 | 37.6 | 0.012 | 0.024 | 0.21711 | 6.12E-01 |
| rs7154911 | 14 | 24643025 | 2.35E-01 | 0.038 | 0.032 | 30.5 | 3.83E-01 | -0.042 | 0.048 | 30.2 | 0.013 | 0.026 | 0.16541 | 6.14E-01 |
| rs9501414 | 6 | 29643490 | 5.61E-01 | 0.056 | 0.096 | 2.3 | 9.75E-01 | 0.005 | 0.147 | 2.1 | 0.041 | 0.080 | 0.76959 | 6.14E-01 |
| rs7356982 | 6 | 26396500 | 8.62E-01 | -0.007 | 0.038 | 16.3 | 5.35E-01 | -0.033 | 0.053 | 21.6 | -0.016 | 0.031 | 0.68846 | 6.14E-01 |
| rs9257932 | 6 | 29626376 | 4.77E-01 | 0.054 | 0.075 | 3.6 | 8.32E-01 | -0.027 | 0.126 | 3.1 | 0.032 | 0.065 | 0.5839 | 6.16E-01 |
| rs2272021 | 19 | 45164732 | 7.92E-01 | 0.015 | 0.056 | 6.8 | 6.20E-01 | 0.039 | 0.080 | 8.5 | 0.023 | 0.046 | 0.79969 | 6.16E-01 |
| rs12294511 | 11 | 5713817 | 9.39E-01 | 0.002 | 0.029 | 45.2 | 4.43E-01 | 0.033 | 0.043 | 44.4 | 0.012 | 0.024 | 0.55651 | 6.17E-01 |
| rs7254892 | 19 | 45389596 | 8.44E-01 | -0.014 | 0.070 | 4.3 | 1.59E-01 | 0.183 | 0.130 | 2.6 | 0.031 | 0.062 | 0.18232 | 6.18E-01 |
| rs157584 | 19 | 45396899 | 8.70E-01 | 0.005 | 0.028 | 47.9 | 5.16E-01 | 0.027 | 0.042 | 45.8 | 0.012 | 0.023 | 0.65274 | 6.19E-01 |
| rs4938706 | 11 | 119539364 | 6.45E-01 | -0.013 | 0.029 | 39.3 | 9.80E-02 | 0.075 | 0.046 | 37.8 | 0.012 | 0.024 | 0.09951 | 6.20E-01 |
| rs2069772 | 4 | 123373133 | 8.09E-01 | -0.008 | 0.032 | 28.2 | 2.30E-01 | 0.055 | 0.046 | 29.0 | 0.013 | 0.026 | 0.26159 | 6.23E-01 |
| rs12202419 | 6 | 26465384 | 6.87E-01 | 0.018 | 0.045 | 11.0 | 7.80E-01 | 0.020 | 0.070 | 10.1 | 0.019 | 0.038 | 0.98634 | 6.24E-01 |
| rs4938717 | 11 | 119576911 | 7.79E-01 | -0.008 | 0.028 | 42.9 | 1.90E-01 | 0.056 | 0.043 | 37.1 | 0.011 | 0.023 | 0.21137 | 6.25E-01 |
| rs1881707 | 3 | 25645421 | 4.80E-01 | 0.022 | 0.032 | 29.5 | 8.73E-01 | -0.007 | 0.045 | 29.0 | 0.013 | 0.026 | 0.59186 | 6.26E-01 |
| rs7260482 | 19 | 45143942 | 4.37E-01 | -0.026 | 0.033 | 25.8 | 7.85E-01 | 0.013 | 0.047 | 28.8 | -0.013 | 0.027 | 0.50184 | 6.32E-01 |
| rs668998 | 3 | 159715551 | 1.60E-01 | -0.040 | 0.028 | 42.4 | 2.18E-01 | 0.052 | 0.042 | 43.3 | -0.011 | 0.024 | 0.07038 | 6.32E-01 |
| rs10417416 | 19 | 45155115 | 9.87E-01 | 0.010 | 0.594 | 0.1 | 5.13E-01 | -0.350 | 0.535 | 0.2 | -0.189 | 0.398 | 0.65245 | 6.35E-01 |
| rs13346041 | 19 | 45140380 | 9.86E-01 | 0.010 | 0.594 | 0.1 | 5.13E-01 | -0.350 | 0.535 | 0.2 | -0.189 | 0.398 | 0.65191 | 6.35E-01 |
| rs7931316 | 11 | 119585681 | 6.13E-01 | -0.014 | 0.027 | 45.2 | 9.92E-02 | 0.070 | 0.043 | 39.8 | 0.011 | 0.023 | 0.09662 | 6.37E-01 |
| rs6671492 | 1 | 161038767 | 2.79E-01 | -0.037 | 0.034 | 22.8 | 4.36E-01 | 0.040 | 0.052 | 23.1 | -0.014 | 0.029 | 0.21216 | 6.37E-01 |
| rs737100 | 19 | 45163451 | 9.86E-01 | 0.010 | 0.594 | 0.1 | 5.19E-01 | -0.346 | 0.536 | 0.2 | -0.186 | 0.398 | 0.65601 | 6.40E-01 |
| rs75546438 | 11 | 119520441 | 3.35E-01 | -0.576 | 0.597 | 0.1 | 6.85E-01 | 0.281 | 0.692 | 0.1 | -0.210 | 0.452 | 0.34854 | 6.42E-01 |
| rs2747420 | 6 | 29642434 | 8.45E-01 | 0.015 | 0.075 | 4.1 | 5.89E-01 | 0.060 | 0.111 | 3.9 | 0.029 | 0.062 | 0.73531 | 6.43E-01 |
| rs931953 | 11 | 119528905 | 6.99E-01 | -0.014 | 0.036 | 21.2 | 8.02E-01 | -0.013 | 0.052 | 22.0 | -0.014 | 0.030 | 0.98748 | 6.45E-01 |
| rs2305742 | 19 | 18191441 | 6.42E-01 | 0.017 | 0.036 | 20.8 | 8.95E-01 | 0.007 | 0.053 | 19.3 | 0.014 | 0.030 | 0.88151 | 6.46E-01 |
| rs1009363 | 11 | 119505032 | 3.22E-01 | 0.028 | 0.028 | 47.2 | 5.19E-01 | -0.027 | 0.042 | 48.7 | 0.011 | 0.023 | 0.2759 | 6.46E-01 |
| rs11265551 | 1 | 161064746 | 2.06E-01 | -0.052 | 0.041 | 13.6 | 2.55E-01 | 0.074 | 0.065 | 12.1 | -0.016 | 0.035 | 0.10094 | 6.47E-01 |
| rs713869 | 22 | 37306899 | 7.47E-01 | 0.011 | 0.033 | 26.5 | 7.33E-01 | 0.017 | 0.051 | 24.6 | 0.013 | 0.027 | 0.9112 | 6.48E-01 |
| rs3760624 | 19 | 45144749 | 5.59E-01 | 0.023 | 0.040 | 15.6 | 9.65E-01 | -0.003 | 0.058 | 15.9 | 0.015 | 0.033 | 0.71252 | 6.48E-01 |
| rs75967483 | 11 | 119556519 | 8.93E-01 | 0.009 | 0.069 | 4.7 | 3.08E-01 | -0.105 | 0.103 | 4.2 | -0.026 | 0.057 | 0.35564 | 6.50E-01 |
| rs12636426 | 3 | 25485721 | 9.69E-01 | -0.002 | 0.053 | 7.8 | 4.01E-01 | 0.063 | 0.075 | 9.3 | 0.019 | 0.043 | 0.47792 | 6.51E-01 |
| rs4394851 | 11 | 119566971 | 8.14E-01 | -0.035 | 0.150 | 0.9 | 2.14E-01 | 0.304 | 0.245 | 0.7 | 0.058 | 0.128 | 0.23684 | 6.52E-01 |
| rs80243646 | 11 | 119566640 | 7.77E-01 | -0.051 | 0.181 | 0.6 | 2.29E-01 | 0.315 | 0.262 | 0.6 | 0.067 | 0.149 | 0.24967 | 6.52E-01 |
| rs11668327 | 19 | 45398633 | 6.53E-01 | 0.017 | 0.037 | 17.4 | 1.51E-01 | -0.077 | 0.054 | 19.3 | -0.014 | 0.031 | 0.1503 | 6.52E-01 |
| rs74334462 | 11 | 119565802 | 7.76E-01 | -0.051 | 0.181 | 0.6 | 2.29E-01 | 0.315 | 0.262 | 0.6 | 0.067 | 0.149 | 0.24929 | 6.53E-01 |
| rs1914926 | 4 | 187015152 | 5.49E-01 | -0.019 | 0.032 | 23.1 | 9.16E-01 | 0.005 | 0.049 | 23.0 | -0.012 | 0.027 | 0.67584 | 6.58E-01 |
| rs2535260 | 6 | 29628883 | 9.93E-01 | 0.000 | 0.036 | 18.1 | 4.30E-01 | 0.042 | 0.053 | 20.1 | 0.013 | 0.030 | 0.50979 | 6.63E-01 |
| rs11217361 | 11 | 119499255 | 6.09E-01 | -0.021 | 0.041 | 13.5 | 1.13E-01 | 0.101 | 0.063 | 12.5 | 0.015 | 0.034 | 0.10681 | 6.65E-01 |
| rs4576834 | 11 | 119542000 | 6.60E-01 | 0.015 | 0.035 | 18.8 | 9.11E-01 | 0.006 | 0.055 | 18.7 | 0.013 | 0.030 | 0.88629 | 6.66E-01 |
| rs7932832 | 11 | 119573524 | 7.19E-01 | -0.010 | 0.029 | 39.7 | 1.88E-01 | 0.057 | 0.044 | 37.9 | 0.010 | 0.024 | 0.19505 | 6.67E-01 |
| rs10405025 | 19 | 45162667 | 8.16E-01 | -0.080 | 0.345 | 0.2 | 6.94E-01 | -0.166 | 0.422 | 0.3 | -0.115 | 0.267 | 0.87459 | 6.68E-01 |
| rs1131936 | 6 | 26394320 | 2.87E-01 | 0.048 | 0.045 | 11.4 | 4.70E-01 | -0.044 | 0.061 | 15.4 | 0.015 | 0.036 | 0.22402 | 6.70E-01 |
| rs3927423 | 6 | 26393487 | 5.63E-01 | 0.027 | 0.046 | 10.6 | 9.16E-01 | -0.007 | 0.068 | 10.2 | 0.016 | 0.038 | 0.68108 | 6.74E-01 |
| rs9468571 | 6 | 29624894 | 8.75E-01 | 0.011 | 0.070 | 4.7 | 6.15E-01 | 0.049 | 0.098 | 4.9 | 0.024 | 0.057 | 0.75085 | 6.75E-01 |
| rs10892433 | 11 | 119573259 | 7.35E-01 | -0.010 | 0.029 | 39.5 | 2.07E-01 | 0.055 | 0.043 | 37.8 | 0.010 | 0.024 | 0.21582 | 6.75E-01 |
| rs2243594 | 21 | 34708061 | 5.48E-01 | 0.018 | 0.029 | 41.4 | 1.11E-01 | -0.067 | 0.042 | 40.2 | -0.010 | 0.024 | 0.09818 | 6.75E-01 |
| rs1800795 | 7 | 22766645 | 9.76E-01 | 0.001 | 0.028 | 44.4 | 4.76E-01 | 0.031 | 0.044 | 42.6 | 0.010 | 0.024 | 0.56184 | 6.78E-01 |
| rs519825 | 19 | 45366779 | 6.62E-01 | 0.013 | 0.029 | 40.8 | 9.29E-01 | 0.004 | 0.043 | 38.9 | 0.010 | 0.024 | 0.86485 | 6.79E-01 |
| rs2770150 | 9 | 120463139 | 6.65E-01 | 0.014 | 0.032 | 27.7 | 1.73E-01 | -0.064 | 0.047 | 24.8 | -0.011 | 0.026 | 0.17005 | 6.80E-01 |
| rs10964995 | 9 | 21439456 | 5.81E-01 | -0.030 | 0.054 | 7.1 | 9.17E-01 | 0.009 | 0.085 | 6.1 | -0.019 | 0.045 | 0.70218 | 6.81E-01 |
| rs2393657 | 6 | 26457688 | 3.90E-01 | -0.062 | 0.072 | 4.3 | 5.79E-02 | 0.191 | 0.100 | 4.8 | 0.024 | 0.059 | 0.04079 | 6.81E-01 |
| rs456298 | 21 | 42836751 | 5.39E-01 | -0.024 | 0.039 | 16.7 | 8.66E-01 | 0.009 | 0.056 | 16.0 | -0.013 | 0.032 | 0.62576 | 6.82E-01 |
| rs2856968 | 21 | 34697981 | 5.43E-01 | 0.018 | 0.029 | 41.4 | 1.11E-01 | -0.067 | 0.042 | 40.2 | -0.010 | 0.024 | 0.09708 | 6.83E-01 |
| rs739718 | 5 | 131873073 | 7.20E-01 | 0.020 | 0.056 | 6.5 | 8.47E-01 | 0.016 | 0.082 | 7.3 | 0.019 | 0.046 | 0.96571 | 6.85E-01 |
| rs7948996 | 11 | 5732222 | 5.61E-01 | -0.020 | 0.035 | 21.4 | 8.85E-01 | 0.007 | 0.052 | 21.1 | -0.012 | 0.029 | 0.65644 | 6.87E-01 |
| rs7924573 | 11 | 119571342 | 7.72E-01 | -0.008 | 0.029 | 38.9 | 2.45E-01 | 0.052 | 0.044 | 37.1 | 0.010 | 0.024 | 0.25775 | 6.90E-01 |
| rs3790569 | 1 | 67836860 | 3.11E-01 | -0.037 | 0.037 | 18.2 | 3.98E-01 | 0.048 | 0.057 | 17.2 | -0.012 | 0.031 | 0.20826 | 6.91E-01 |
| rs77241309 | 19 | 45349177 | 7.50E-01 | -0.014 | 0.044 | 11.4 | 8.22E-01 | -0.015 | 0.067 | 10.6 | -0.014 | 0.037 | 0.99004 | 6.97E-01 |
| rs2393664 | 6 | 26460254 | 9.60E-01 | 0.002 | 0.038 | 16.3 | 5.53E-01 | 0.031 | 0.052 | 20.7 | 0.012 | 0.031 | 0.65193 | 6.97E-01 |
| rs4459318 | 11 | 119541778 | 5.59E-01 | -0.017 | 0.029 | 40.2 | 1.02E-01 | 0.073 | 0.045 | 38.7 | 0.009 | 0.024 | 0.09023 | 6.98E-01 |
| rs73571271 | 11 | 119509485 | 6.80E-01 | -0.174 | 0.421 | 0.1 | 9.51E-01 | -0.043 | 0.692 | 0.1 | -0.139 | 0.360 | 0.8717 | 7.00E-01 |
| rs999259 | 21 | 34666492 | 6.31E-01 | 0.014 | 0.030 | 48.3 | 1.78E-01 | -0.057 | 0.042 | 46.9 | -0.009 | 0.024 | 0.16789 | 7.01E-01 |
| rs6822014 | 4 | 186985201 | 5.65E-01 | 0.020 | 0.035 | 19.2 | 8.51E-01 | -0.010 | 0.053 | 20.0 | 0.011 | 0.029 | 0.63497 | 7.08E-01 |
| rs7945395 | 11 | 119569737 | 6.83E-01 | -0.012 | 0.029 | 39.8 | 1.95E-01 | 0.057 | 0.044 | 37.8 | 0.009 | 0.024 | 0.191 | 7.08E-01 |
| rs9467759 | 6 | 26464472 | 1.85E-01 | 0.056 | 0.042 | 13.1 | 2.49E-01 | -0.065 | 0.057 | 16.9 | 0.013 | 0.034 | 0.08599 | 7.09E-01 |
| rs56210316 | 19 | 45170097 | 5.18E-01 | 0.059 | 0.092 | 2.3 | 8.17E-01 | -0.027 | 0.118 | 3.4 | 0.027 | 0.072 | 0.56243 | 7.12E-01 |
| rs11265550 | 1 | 161060390 | 5.66E-01 | -0.017 | 0.029 | 46.8 | 8.47E-01 | 0.008 | 0.043 | 45.3 | -0.009 | 0.024 | 0.62762 | 7.16E-01 |
| rs2228145 | 1 | 154426970 | 3.64E-01 | 0.027 | 0.030 | 37.5 | 5.16E-01 | -0.027 | 0.042 | 39.8 | 0.009 | 0.024 | 0.29158 | 7.16E-01 |
| rs1012334 | 21 | 34713751 | 5.25E-01 | 0.018 | 0.029 | 46.9 | 1.16E-01 | -0.067 | 0.042 | 46.0 | -0.009 | 0.024 | 0.09711 | 7.17E-01 |
| rs2045386 | 19 | 18182266 | 1.43E-01 | -0.042 | 0.029 | 42.5 | 1.16E-01 | 0.069 | 0.044 | 40.8 | -0.009 | 0.024 | 0.03386 | 7.19E-01 |
| rs41267929 | 6 | 26468994 | 6.16E-01 | -0.031 | 0.061 | 5.4 | 9.24E-01 | 0.008 | 0.087 | 6.4 | -0.018 | 0.050 | 0.71467 | 7.21E-01 |
| rs4412763 | 11 | 119507626 | 3.70E-01 | -0.310 | 0.346 | 0.2 | 5.16E-01 | 0.317 | 0.488 | 0.2 | -0.100 | 0.282 | 0.29439 | 7.22E-01 |
| rs11825206 | 11 | 119570128 | 6.49E-01 | -0.013 | 0.029 | 39.9 | 1.88E-01 | 0.057 | 0.044 | 37.9 | 0.008 | 0.024 | 0.17724 | 7.27E-01 |
| rs4938699 | 11 | 119510937 | 9.23E-01 | 0.005 | 0.052 | 8.1 | 4.28E-01 | -0.064 | 0.080 | 7.7 | -0.015 | 0.043 | 0.47212 | 7.27E-01 |
| rs7940667 | 11 | 119510644 | 9.23E-01 | 0.005 | 0.052 | 8.1 | 4.28E-01 | -0.064 | 0.080 | 7.7 | -0.015 | 0.043 | 0.47212 | 7.27E-01 |
| rs57537848 | 19 | 45354044 | 9.36E-01 | -0.002 | 0.028 | 46.0 | 6.35E-01 | -0.020 | 0.041 | 46.1 | -0.008 | 0.023 | 0.72925 | 7.37E-01 |
| rs12118313 | 1 | 161037979 | 3.53E-01 | -0.031 | 0.034 | 23.2 | 4.38E-01 | 0.038 | 0.049 | 23.5 | -0.009 | 0.028 | 0.24438 | 7.38E-01 |
| rs203709 | 19 | 45161566 | 5.46E-01 | -0.020 | 0.033 | 25.5 | 7.75E-01 | 0.013 | 0.047 | 28.9 | -0.009 | 0.027 | 0.56235 | 7.40E-01 |
| rs10946822 | 6 | 26414678 | 8.52E-01 | 0.008 | 0.043 | 12.8 | 7.59E-01 | 0.019 | 0.062 | 12.6 | 0.012 | 0.035 | 0.88453 | 7.43E-01 |
| rs1424860 | 9 | 21073945 | 7.76E-01 | -0.012 | 0.042 | 13.3 | 8.76E-01 | -0.010 | 0.062 | 12.9 | -0.011 | 0.034 | 0.97654 | 7.46E-01 |
| rs73736251 | 6 | 26395165 | 7.10E-01 | -0.034 | 0.091 | 2.5 | 2.08E-01 | 0.192 | 0.153 | 1.9 | 0.025 | 0.078 | 0.20259 | 7.46E-01 |
| rs10790331 | 11 | 119552057 | 6.98E-01 | -0.011 | 0.028 | 40.4 | 2.30E-01 | 0.054 | 0.045 | 38.5 | 0.008 | 0.024 | 0.22156 | 7.52E-01 |
| rs1293749 | 12 | 113442257 | 6.25E-01 | 0.015 | 0.031 | 30.6 | 8.80E-01 | -0.007 | 0.045 | 30.4 | 0.008 | 0.025 | 0.68712 | 7.52E-01 |
| rs1110166 | 11 | 119571975 | 7.01E-01 | -0.011 | 0.029 | 39.3 | 2.49E-01 | 0.051 | 0.044 | 37.4 | 0.008 | 0.024 | 0.24023 | 7.53E-01 |
| rs6793694 | 3 | 25491968 | 6.16E-01 | -0.015 | 0.029 | 38.7 | 8.36E-01 | 0.009 | 0.044 | 39.3 | -0.007 | 0.025 | 0.65297 | 7.61E-01 |
| rs10410651 | 19 | 45160896 | 5.78E-01 | -0.018 | 0.033 | 25.3 | 7.91E-01 | 0.012 | 0.047 | 28.8 | -0.008 | 0.027 | 0.59135 | 7.62E-01 |
| rs6589769 | 11 | 119574060 | 6.46E-01 | -0.013 | 0.029 | 40.0 | 2.18E-01 | 0.054 | 0.043 | 38.2 | 0.007 | 0.024 | 0.20001 | 7.62E-01 |
| rs10811469 | 9 | 21086340 | 7.85E-01 | -0.011 | 0.042 | 13.3 | 8.93E-01 | -0.008 | 0.062 | 12.8 | -0.010 | 0.035 | 0.96845 | 7.63E-01 |
| rs10892441 | 11 | 119586418 | 4.50E-01 | -0.021 | 0.028 | 42.1 | 8.63E-02 | 0.074 | 0.043 | 37.5 | 0.007 | 0.023 | 0.06395 | 7.64E-01 |
| rs157588 | 19 | 45398264 | 8.66E-01 | 0.005 | 0.028 | 47.5 | 7.76E-01 | 0.012 | 0.041 | 45.8 | 0.007 | 0.023 | 0.88996 | 7.64E-01 |
| rs11590097 | 1 | 161056989 | 3.03E-01 | -0.044 | 0.042 | 12.7 | 2.95E-01 | 0.069 | 0.066 | 11.7 | -0.011 | 0.036 | 0.14991 | 7.64E-01 |
| rs2179 | 11 | 5723107 | 9.48E-01 | -0.002 | 0.031 | 29.8 | 6.60E-01 | -0.021 | 0.048 | 28.1 | -0.008 | 0.026 | 0.73938 | 7.68E-01 |
| rs35301288 | 11 | 119578224 | 4.21E-01 | -0.022 | 0.028 | 42.2 | 7.54E-02 | 0.077 | 0.043 | 37.2 | 0.007 | 0.023 | 0.05315 | 7.74E-01 |
| rs10892425 | 11 | 119516100 | 6.42E-01 | 0.026 | 0.056 | 6.6 | 2.11E-01 | -0.109 | 0.087 | 6.3 | -0.014 | 0.047 | 0.1923 | 7.75E-01 |
| rs4803763 | 19 | 45357291 | 9.87E-01 | -0.001 | 0.033 | 26.9 | 6.04E-01 | 0.025 | 0.048 | 25.5 | 0.007 | 0.027 | 0.66133 | 7.81E-01 |
| rs4582984 | 11 | 119499014 | 5.10E-01 | 0.021 | 0.032 | 26.0 | 6.31E-01 | -0.022 | 0.047 | 25.4 | 0.007 | 0.026 | 0.44319 | 7.82E-01 |
| rs12273351 | 11 | 119503402 | 1.67E-01 | -0.041 | 0.030 | 40.3 | 1.38E-01 | 0.062 | 0.042 | 40.0 | -0.007 | 0.024 | 0.04437 | 7.82E-01 |
| rs12270510 | 11 | 119506018 | 8.69E-01 | 0.008 | 0.049 | 10.8 | 4.56E-01 | -0.055 | 0.074 | 10.1 | -0.011 | 0.041 | 0.47605 | 7.84E-01 |
| rs2089648 | 11 | 119512770 | 5.75E-01 | 0.033 | 0.059 | 6.0 | 1.77E-01 | -0.122 | 0.090 | 5.7 | -0.013 | 0.049 | 0.15033 | 7.87E-01 |
| rs73048293 | 19 | 45340736 | 8.30E-01 | -0.007 | 0.031 | 30.7 | 4.22E-01 | 0.037 | 0.047 | 28.3 | 0.007 | 0.026 | 0.43103 | 7.87E-01 |
| rs4936489 | 11 | 119506642 | 9.95E-01 | 0.001 | 0.226 | 0.4 | 5.15E-01 | -0.320 | 0.491 | 0.2 | -0.055 | 0.205 | 0.55223 | 7.90E-01 |
| rs77910615 | 11 | 119558235 | 6.96E-01 | -0.095 | 0.243 | 0.3 | 7.50E-01 | 0.220 | 0.690 | 0.1 | -0.060 | 0.229 | 0.66694 | 7.92E-01 |
| rs11217406 | 11 | 119564180 | 6.97E-01 | -0.095 | 0.243 | 0.3 | 7.53E-01 | 0.217 | 0.690 | 0.1 | -0.060 | 0.229 | 0.66973 | 7.93E-01 |
| rs11668861 | 19 | 45380970 | 3.60E-01 | -0.026 | 0.029 | 45.6 | 3.72E-01 | 0.038 | 0.043 | 44.4 | -0.006 | 0.024 | 0.21038 | 7.93E-01 |
| rs11217403 | 11 | 119563617 | 6.97E-01 | -0.095 | 0.243 | 0.3 | 7.50E-01 | 0.220 | 0.690 | 0.1 | -0.060 | 0.229 | 0.66748 | 7.94E-01 |
| rs57088022 | 11 | 119562522 | 6.97E-01 | -0.095 | 0.243 | 0.3 | 7.50E-01 | 0.220 | 0.690 | 0.1 | -0.060 | 0.229 | 0.66748 | 7.94E-01 |
| rs78803563 | 11 | 119563386 | 6.97E-01 | -0.095 | 0.243 | 0.3 | 7.50E-01 | 0.220 | 0.690 | 0.1 | -0.060 | 0.229 | 0.66748 | 7.94E-01 |
| rs2436474 | 19 | 45362269 | 8.36E-01 | 0.006 | 0.029 | 40.4 | 8.77E-01 | 0.007 | 0.043 | 38.8 | 0.006 | 0.024 | 0.98949 | 7.97E-01 |
| rs7549250 | 1 | 154404336 | 2.74E-01 | -0.032 | 0.029 | 44.2 | 2.53E-01 | 0.048 | 0.042 | 43.6 | -0.006 | 0.024 | 0.11803 | 7.97E-01 |
| rs34163098 | 11 | 119509118 | 2.11E-01 | -0.037 | 0.029 | 40.1 | 1.67E-01 | 0.059 | 0.042 | 39.5 | -0.006 | 0.024 | 0.06431 | 8.04E-01 |
| rs2834211 | 21 | 34789808 | 4.33E-01 | -0.038 | 0.048 | 9.9 | 1.29E-01 | 0.103 | 0.067 | 10.9 | 0.010 | 0.039 | 0.09016 | 8.07E-01 |
| rs4921466 | 5 | 158732772 | 9.21E-01 | -0.005 | 0.050 | 9.6 | 6.00E-01 | 0.034 | 0.065 | 11.8 | 0.010 | 0.040 | 0.6344 | 8.08E-01 |
| rs7124934 | 11 | 119564781 | 9.05E-01 | -0.003 | 0.028 | 44.4 | 5.24E-01 | 0.028 | 0.044 | 42.3 | 0.006 | 0.023 | 0.54696 | 8.09E-01 |
| rs6912853 | 6 | 26401438 | 7.36E-01 | -0.013 | 0.039 | 15.8 | 9.48E-01 | 0.004 | 0.055 | 16.8 | -0.008 | 0.032 | 0.80466 | 8.11E-01 |
| rs9303286 | 17 | 38500928 | 9.66E-01 | 0.002 | 0.046 | 10.4 | 6.19E-01 | -0.036 | 0.073 | 9.9 | -0.009 | 0.039 | 0.65668 | 8.20E-01 |
| rs4804806 | 19 | 7816625 | 7.21E-01 | -0.010 | 0.029 | 34.9 | 3.41E-01 | 0.043 | 0.045 | 37.4 | 0.005 | 0.024 | 0.31963 | 8.30E-01 |
| rs10813829 | 9 | 32517175 | 3.95E-01 | 0.025 | 0.030 | 36.0 | 3.72E-01 | -0.040 | 0.045 | 37.6 | 0.005 | 0.025 | 0.22421 | 8.31E-01 |
| rs73229194 | 3 | 110857552 | 6.77E-01 | -0.020 | 0.049 | 9.7 | 8.26E-01 | 0.015 | 0.069 | 10.9 | -0.008 | 0.040 | 0.67404 | 8.31E-01 |
| rs76161919 | 6 | 26476380 | 6.87E-02 | -0.344 | 0.189 | 0.6 | 1.38E-01 | 0.275 | 0.185 | 1.3 | -0.028 | 0.132 | 0.01916 | 8.33E-01 |
| rs10807008 | 6 | 26405264 | 5.36E-01 | 0.030 | 0.048 | 9.7 | 6.16E-01 | -0.034 | 0.067 | 11.6 | 0.008 | 0.039 | 0.44195 | 8.34E-01 |
| rs906827 | 11 | 119514129 | 5.69E-01 | 0.032 | 0.057 | 6.5 | 2.11E-01 | -0.109 | 0.087 | 6.3 | -0.010 | 0.048 | 0.17427 | 8.35E-01 |
| rs7129848 | 11 | 119566228 | 9.78E-01 | 0.001 | 0.034 | 23.9 | 7.47E-01 | 0.016 | 0.050 | 23.9 | 0.006 | 0.028 | 0.80123 | 8.39E-01 |
| rs6901118 | 6 | 26399586 | 7.24E-01 | 0.024 | 0.069 | 4.2 | 4.70E-01 | -0.058 | 0.081 | 7.5 | -0.011 | 0.052 | 0.43628 | 8.39E-01 |
| rs79519651 | 6 | 26478088 | 2.35E-01 | 0.705 | 0.593 | 0.1 | 5.56E-01 | -0.246 | 0.417 | 0.3 | 0.069 | 0.341 | 0.19002 | 8.40E-01 |
| rs7932236 | 11 | 119515549 | 6.43E-01 | 0.124 | 0.267 | 0.3 | 2.92E-01 | -0.421 | 0.400 | 0.3 | -0.045 | 0.222 | 0.2569 | 8.41E-01 |
| rs12410477 | 1 | 161056214 | 5.71E-01 | 0.027 | 0.048 | 10.0 | 6.58E-01 | -0.029 | 0.066 | 12.0 | 0.008 | 0.039 | 0.48928 | 8.43E-01 |
| rs4712990 | 6 | 26413744 | 5.18E-01 | 0.031 | 0.048 | 9.9 | 5.67E-01 | -0.038 | 0.067 | 11.6 | 0.007 | 0.039 | 0.39922 | 8.48E-01 |
| rs75354183 | 3 | 110836171 | 9.66E-01 | -0.007 | 0.166 | 0.7 | 7.98E-01 | -0.055 | 0.213 | 1.0 | -0.025 | 0.131 | 0.86043 | 8.48E-01 |
| rs2229857 | 1 | 154573967 | 9.73E-01 | -0.001 | 0.031 | 28.2 | 7.01E-01 | 0.017 | 0.044 | 30.4 | 0.005 | 0.025 | 0.73734 | 8.49E-01 |
| rs12610257 | 19 | 45363392 | 7.84E-01 | -0.010 | 0.037 | 17.8 | 4.52E-01 | 0.042 | 0.056 | 16.9 | 0.006 | 0.031 | 0.43582 | 8.51E-01 |
| rs2893856 | 6 | 26460721 | 4.68E-01 | 0.031 | 0.043 | 12.1 | 5.04E-01 | -0.039 | 0.058 | 15.8 | 0.006 | 0.034 | 0.3322 | 8.52E-01 |
| rs3846850 | 6 | 26449618 | 3.32E-01 | 0.335 | 0.344 | 0.2 | 1.16E-01 | -1.119 | 0.710 | 0.1 | 0.058 | 0.310 | 0.06556 | 8.52E-01 |
| rs10484440 | 6 | 26453742 | 5.88E-01 | -0.023 | 0.043 | 13.0 | 2.62E-01 | 0.071 | 0.063 | 13.0 | 0.007 | 0.036 | 0.21709 | 8.54E-01 |
| rs3024496 | 1 | 206941864 | 9.24E-01 | 0.003 | 0.028 | 48.1 | 6.35E-01 | -0.020 | 0.043 | 47.1 | -0.004 | 0.024 | 0.65319 | 8.56E-01 |
| rs228945 | 22 | 37525880 | 7.81E-01 | 0.009 | 0.031 | 30.4 | 4.55E-01 | -0.035 | 0.047 | 28.7 | -0.005 | 0.026 | 0.43747 | 8.58E-01 |
| rs382634 | 19 | 18187562 | 8.08E-01 | 0.007 | 0.029 | 33.4 | 9.68E-01 | -0.002 | 0.043 | 30.9 | 0.004 | 0.024 | 0.86548 | 8.58E-01 |
| rs443099 | 21 | 42743327 | 9.06E-01 | -0.003 | 0.029 | 38.2 | 8.88E-01 | -0.006 | 0.043 | 39.0 | -0.004 | 0.024 | 0.9578 | 8.60E-01 |
| rs10892424 | 11 | 119515046 | 5.99E-01 | 0.030 | 0.056 | 6.5 | 2.54E-01 | -0.100 | 0.088 | 6.1 | -0.008 | 0.048 | 0.21316 | 8.61E-01 |
| rs59438701 | 6 | 26412527 | 5.87E-01 | -0.026 | 0.048 | 11.5 | 6.75E-01 | 0.026 | 0.062 | 12.5 | -0.007 | 0.038 | 0.50607 | 8.63E-01 |
| rs962859 | 21 | 34648123 | 9.83E-01 | -0.001 | 0.030 | 42.7 | 7.54E-01 | 0.013 | 0.040 | 42.9 | 0.004 | 0.024 | 0.79147 | 8.65E-01 |
| rs4633144 | 9 | 32504294 | 4.15E-01 | 0.024 | 0.029 | 36.4 | 3.46E-01 | -0.042 | 0.045 | 37.7 | 0.004 | 0.025 | 0.21636 | 8.67E-01 |
| rs7122134 | 11 | 119547005 | 5.77E-01 | -0.016 | 0.028 | 45.1 | 5.64E-01 | 0.025 | 0.044 | 42.5 | -0.004 | 0.023 | 0.43176 | 8.72E-01 |
| rs12162222 | 19 | 45348522 | 6.11E-01 | -0.016 | 0.032 | 28.7 | 6.48E-01 | 0.021 | 0.047 | 27.3 | -0.004 | 0.026 | 0.50569 | 8.74E-01 |
| rs12150884 | 19 | 18179973 | 9.12E-01 | 0.004 | 0.036 | 20.6 | 9.11E-01 | 0.006 | 0.053 | 19.4 | 0.005 | 0.030 | 0.97486 | 8.77E-01 |
| rs17777460 | 15 | 89173556 | 5.82E-01 | -0.025 | 0.045 | 11.2 | 5.72E-01 | 0.040 | 0.070 | 10.8 | -0.006 | 0.038 | 0.43874 | 8.78E-01 |
| rs4712993 | 6 | 26416399 | 8.16E-01 | 0.010 | 0.042 | 13.2 | 5.65E-01 | -0.033 | 0.057 | 17.1 | -0.005 | 0.033 | 0.54702 | 8.79E-01 |
| rs12946680 | 17 | 38480657 | 6.89E-01 | 0.017 | 0.042 | 12.9 | 7.22E-01 | -0.024 | 0.067 | 11.6 | 0.005 | 0.036 | 0.6066 | 8.81E-01 |
| rs12462573 | 19 | 45359706 | 8.35E-01 | -0.007 | 0.033 | 26.7 | 5.70E-01 | 0.027 | 0.048 | 25.4 | 0.004 | 0.027 | 0.55688 | 8.82E-01 |
| rs56161420 | 6 | 26405835 | 5.37E-01 | 0.025 | 0.041 | 13.6 | 5.50E-01 | -0.033 | 0.056 | 17.6 | 0.005 | 0.033 | 0.3968 | 8.84E-01 |
| rs1127317 | 1 | 154556040 | 9.51E-01 | -0.002 | 0.031 | 27.9 | 7.29E-01 | 0.016 | 0.045 | 29.8 | 0.004 | 0.025 | 0.74791 | 8.85E-01 |
| rs3902051 | 6 | 26403703 | 7.47E-01 | 0.015 | 0.046 | 10.5 | 4.84E-01 | -0.045 | 0.065 | 12.9 | -0.005 | 0.037 | 0.44859 | 8.85E-01 |
| rs3903661 | 6 | 26421876 | 5.80E-01 | 0.023 | 0.041 | 13.3 | 6.00E-01 | -0.030 | 0.057 | 17.0 | 0.005 | 0.033 | 0.45287 | 8.88E-01 |
| rs56190518 | 6 | 26413975 | 7.56E-01 | 0.013 | 0.041 | 13.6 | 5.08E-01 | -0.038 | 0.057 | 17.2 | -0.005 | 0.033 | 0.47184 | 8.91E-01 |
| rs6920297 | 6 | 29618032 | 9.61E-01 | -0.012 | 0.244 | 0.3 | 8.93E-01 | -0.026 | 0.191 | 1.3 | -0.020 | 0.150 | 0.96466 | 8.91E-01 |
| rs6456722 | 6 | 26421276 | 5.57E-01 | 0.024 | 0.041 | 13.4 | 5.65E-01 | -0.033 | 0.057 | 17.1 | 0.005 | 0.033 | 0.41681 | 8.92E-01 |
| rs2546893 | 5 | 158755960 | 7.38E-01 | 0.010 | 0.029 | 48.4 | 8.09E-01 | -0.010 | 0.040 | 48.2 | 0.003 | 0.024 | 0.69452 | 8.97E-01 |
| rs11622031 | 14 | 24624191 | 4.52E-01 | -0.022 | 0.029 | 49.9 | 1.87E-01 | -0.056 | 0.042 | 48.6 | -0.003 | 0.024 | 0.12958 | 9.00E-01 |
| rs12976908 | 19 | 18174377 | 5.51E-01 | 0.018 | 0.029 | 38.5 | 5.07E-01 | -0.029 | 0.044 | 37.5 | 0.003 | 0.024 | 0.3772 | 9.00E-01 |
| rs16832617 | 1 | 161037857 | 7.29E-01 | 0.015 | 0.044 | 11.8 | 8.04E-01 | -0.014 | 0.057 | 15.1 | 0.004 | 0.035 | 0.68344 | 9.01E-01 |
| rs1567375 | 11 | 119502477 | 2.54E-01 | -0.033 | 0.029 | 42.2 | 1.57E-01 | 0.059 | 0.041 | 40.9 | -0.003 | 0.024 | 0.06936 | 9.05E-01 |
| rs7927749 | 11 | 119585051 | 4.84E-01 | -0.295 | 0.421 | 0.1 | 5.26E-01 | 0.310 | 0.488 | 0.2 | -0.037 | 0.319 | 0.3486 | 9.07E-01 |
| rs10892430 | 11 | 119559027 | 9.98E-01 | 0.001 | 0.266 | 0.3 | 7.50E-01 | 0.220 | 0.690 | 0.1 | 0.029 | 0.248 | 0.76704 | 9.07E-01 |
| rs1467050 | 11 | 119529365 | 4.12E-01 | 0.047 | 0.057 | 6.4 | 2.75E-01 | -0.099 | 0.090 | 5.9 | 0.005 | 0.048 | 0.17321 | 9.12E-01 |
| rs28807203 | 19 | 45173951 | 9.71E-01 | -0.003 | 0.073 | 3.8 | 8.39E-01 | 0.018 | 0.089 | 6.1 | 0.006 | 0.056 | 0.85658 | 9.20E-01 |
| rs4252279 | 11 | 117867187 | 7.33E-01 | -0.016 | 0.046 | 10.6 | 4.99E-01 | 0.046 | 0.068 | 11.1 | 0.004 | 0.038 | 0.45219 | 9.23E-01 |
| rs3745150 | 19 | 45385759 | 8.79E-01 | 0.004 | 0.029 | 39.3 | 9.51E-01 | -0.003 | 0.043 | 38.8 | 0.002 | 0.024 | 0.89204 | 9.26E-01 |
| rs11217428 | 11 | 119592989 | 3.54E-01 | -0.551 | 0.594 | 0.1 | 5.21E-01 | 0.313 | 0.487 | 0.2 | -0.034 | 0.377 | 0.26124 | 9.27E-01 |
| rs3846849 | 6 | 26449436 | 5.53E-01 | 0.025 | 0.041 | 13.4 | 5.11E-01 | -0.037 | 0.057 | 17.0 | 0.003 | 0.033 | 0.37807 | 9.27E-01 |
| rs10983754 | 9 | 120458607 | 2.25E-01 | -0.072 | 0.059 | 6.2 | 7.96E-02 | 0.137 | 0.078 | 8.0 | 0.004 | 0.047 | 0.0329 | 9.27E-01 |
| rs952146 | 1 | 154368928 | 5.22E-01 | 0.018 | 0.029 | 39.5 | 2.78E-01 | -0.045 | 0.042 | 38.4 | -0.002 | 0.024 | 0.20854 | 9.28E-01 |
| rs7544381 | 1 | 67774293 | 3.68E-01 | -0.027 | 0.030 | 32.3 | 2.35E-01 | 0.054 | 0.045 | 32.3 | -0.002 | 0.025 | 0.13678 | 9.29E-01 |
| rs29228 | 6 | 29623739 | 5.94E-01 | 0.019 | 0.036 | 20.4 | 5.43E-01 | -0.031 | 0.052 | 21.6 | 0.003 | 0.030 | 0.42134 | 9.30E-01 |
| rs2239749 | 22 | 37331305 | 1.84E-01 | -0.039 | 0.029 | 46.6 | 7.69E-02 | 0.075 | 0.042 | 46.0 | -0.002 | 0.024 | 0.02677 | 9.30E-01 |
| rs12401309 | 1 | 161051558 | 8.12E-01 | -0.012 | 0.052 | 8.1 | 8.29E-01 | 0.018 | 0.081 | 8.1 | -0.004 | 0.044 | 0.75606 | 9.33E-01 |
| rs11217408 | 11 | 119564483 | 3.91E-01 | -0.129 | 0.151 | 0.9 | 2.17E-01 | 0.302 | 0.245 | 0.7 | -0.010 | 0.128 | 0.13288 | 9.35E-01 |
| rs79178970 | 11 | 119508845 | 3.33E-01 | -0.577 | 0.596 | 0.1 | 3.22E-01 | 0.678 | 0.683 | 0.1 | -0.035 | 0.449 | 0.16608 | 9.37E-01 |
| rs3892376 | 1 | 161053773 | 7.06E-01 | 0.016 | 0.042 | 13.3 | 6.64E-01 | -0.028 | 0.064 | 12.3 | 0.003 | 0.035 | 0.56828 | 9.41E-01 |
| rs11217404 | 11 | 119563681 | 3.97E-01 | -0.128 | 0.150 | 0.9 | 2.17E-01 | 0.302 | 0.245 | 0.7 | -0.009 | 0.128 | 0.13424 | 9.41E-01 |
| rs168681 | 5 | 131402450 | 8.20E-01 | 0.007 | 0.029 | 42.2 | 8.33E-01 | -0.009 | 0.043 | 39.0 | 0.002 | 0.024 | 0.76321 | 9.42E-01 |
| rs1411614 | 1 | 161057909 | 7.93E-01 | 0.009 | 0.033 | 27.0 | 6.10E-01 | -0.025 | 0.049 | 27.0 | -0.002 | 0.027 | 0.56849 | 9.45E-01 |
| rs6765133 | 3 | 110794998 | 8.78E-01 | 0.024 | 0.155 | 0.9 | 9.41E-01 | -0.014 | 0.182 | 1.4 | 0.008 | 0.118 | 0.87616 | 9.46E-01 |
| rs7616280 | 3 | 110833671 | 3.89E-01 | -0.068 | 0.079 | 3.6 | 1.67E-01 | 0.160 | 0.115 | 3.6 | 0.004 | 0.065 | 0.10321 | 9.47E-01 |
| rs1466435 | 19 | 45342815 | 6.07E-01 | 0.032 | 0.062 | 5.2 | 2.59E-01 | -0.136 | 0.120 | 3.2 | -0.003 | 0.055 | 0.2152 | 9.52E-01 |
| rs7642664 | 3 | 110788941 | 4.11E-01 | -0.065 | 0.079 | 3.6 | 1.87E-01 | 0.154 | 0.117 | 3.5 | 0.004 | 0.065 | 0.12004 | 9.54E-01 |
| rs2292293 | 11 | 119548698 | 3.74E-01 | -0.026 | 0.029 | 38.7 | 2.03E-01 | 0.058 | 0.046 | 39.1 | -0.001 | 0.025 | 0.12061 | 9.54E-01 |
| rs12211872 | 6 | 26472265 | 5.86E-01 | 0.027 | 0.050 | 9.1 | 5.23E-01 | -0.043 | 0.067 | 11.3 | 0.002 | 0.040 | 0.40166 | 9.54E-01 |
| rs1467051 | 11 | 119568301 | 6.20E-01 | -0.014 | 0.029 | 40.2 | 3.91E-01 | 0.038 | 0.044 | 39.1 | 0.001 | 0.024 | 0.32233 | 9.55E-01 |
| rs873133 | 3 | 110789199 | 5.70E-01 | 0.053 | 0.094 | 2.4 | 5.22E-01 | -0.077 | 0.121 | 3.1 | 0.004 | 0.074 | 0.39255 | 9.56E-01 |
| rs11575934 | 19 | 18186618 | 6.76E-01 | 0.013 | 0.030 | 32.0 | 5.97E-01 | -0.024 | 0.046 | 29.4 | 0.001 | 0.025 | 0.50149 | 9.57E-01 |
| rs73050216 | 19 | 45367502 | 6.23E-01 | -0.018 | 0.037 | 18.0 | 4.01E-01 | 0.047 | 0.056 | 16.9 | 0.002 | 0.031 | 0.33042 | 9.58E-01 |
| rs10946825 | 6 | 26416866 | 6.29E-01 | 0.020 | 0.041 | 13.3 | 5.65E-01 | -0.033 | 0.057 | 17.1 | 0.002 | 0.033 | 0.45292 | 9.59E-01 |
| rs11217378 | 11 | 119530766 | 5.90E-01 | 0.186 | 0.344 | 0.2 | 4.98E-01 | -0.331 | 0.488 | 0.2 | 0.014 | 0.281 | 0.38737 | 9.59E-01 |
| rs10892423 | 11 | 119506483 | 4.37E-01 | 0.022 | 0.028 | 46.8 | 2.75E-01 | -0.047 | 0.043 | 49.4 | 0.001 | 0.024 | 0.18034 | 9.59E-01 |
| rs5030728 | 9 | 120474282 | 7.21E-01 | 0.011 | 0.031 | 30.0 | 5.41E-01 | -0.028 | 0.046 | 27.5 | -0.001 | 0.026 | 0.47981 | 9.61E-01 |
| rs2284552 | 21 | 34644082 | 9.71E-01 | -0.001 | 0.035 | 20.9 | 9.76E-01 | -0.002 | 0.052 | 19.0 | -0.001 | 0.029 | 0.99654 | 9.63E-01 |
| rs4938697 | 11 | 119507468 | 2.94E-01 | -0.030 | 0.029 | 43.3 | 1.52E-01 | 0.060 | 0.042 | 41.8 | -0.001 | 0.024 | 0.07581 | 9.63E-01 |
| rs3852859 | 19 | 45379309 | 4.05E-01 | -0.031 | 0.037 | 18.7 | 2.60E-01 | 0.060 | 0.053 | 19.6 | -0.001 | 0.030 | 0.16091 | 9.63E-01 |
| rs8038865 | 15 | 89181102 | 3.48E-01 | -0.029 | 0.031 | 28.2 | 1.18E-01 | 0.077 | 0.049 | 25.8 | 0.001 | 0.026 | 0.06812 | 9.64E-01 |
| rs6476363 | 9 | 32491240 | 6.15E-01 | 0.014 | 0.028 | 40.4 | 4.01E-01 | -0.036 | 0.043 | 42.2 | -0.001 | 0.024 | 0.32733 | 9.67E-01 |
| rs6929846 | 6 | 26458265 | 7.23E-01 | 0.014 | 0.040 | 15.1 | 5.96E-01 | -0.028 | 0.052 | 20.4 | -0.001 | 0.032 | 0.52387 | 9.69E-01 |
| rs7950059 | 11 | 119565495 | 5.65E-01 | -0.017 | 0.029 | 41.2 | 3.48E-01 | 0.041 | 0.043 | 40.0 | 0.001 | 0.024 | 0.27061 | 9.70E-01 |
| rs2535246 | 6 | 29636409 | 6.81E-01 | -0.016 | 0.038 | 16.4 | 5.91E-01 | 0.030 | 0.055 | 17.7 | -0.001 | 0.031 | 0.49907 | 9.71E-01 |
| rs2075649 | 19 | 45395330 | 7.02E-01 | -0.011 | 0.029 | 38.9 | 5.38E-01 | 0.026 | 0.043 | 38.4 | 0.001 | 0.024 | 0.46818 | 9.75E-01 |
| rs873132 | 3 | 110789092 | 5.86E-01 | 0.052 | 0.095 | 2.4 | 5.22E-01 | -0.077 | 0.121 | 3.1 | 0.002 | 0.075 | 0.40043 | 9.75E-01 |
| rs7248637 | 19 | 7807027 | 7.06E-01 | -0.019 | 0.051 | 8.9 | 6.44E-01 | 0.032 | 0.069 | 10.8 | -0.001 | 0.041 | 0.55102 | 9.76E-01 |
| rs3852861 | 19 | 45383061 | 6.57E-01 | -0.013 | 0.029 | 39.4 | 5.35E-01 | 0.028 | 0.045 | 40.7 | -0.001 | 0.025 | 0.44594 | 9.77E-01 |
| rs28483039 | 19 | 45149235 | 9.29E-01 | -0.003 | 0.034 | 23.8 | 9.35E-01 | 0.004 | 0.048 | 26.2 | -0.001 | 0.028 | 0.90594 | 9.79E-01 |
| rs11064145 | 12 | 6455098 | 4.07E-01 | -0.024 | 0.029 | 44.8 | 2.43E-01 | 0.050 | 0.043 | 44.8 | -0.001 | 0.024 | 0.15178 | 9.79E-01 |
| rs4329505 | 1 | 154432420 | 9.82E-01 | 0.001 | 0.038 | 17.2 | 9.40E-01 | -0.004 | 0.057 | 15.3 | -0.001 | 0.032 | 0.94039 | 9.81E-01 |
| rs12208272 | 6 | 26477640 | 5.79E-01 | 0.028 | 0.050 | 8.9 | 4.77E-01 | -0.048 | 0.067 | 11.2 | 0.001 | 0.040 | 0.36714 | 9.83E-01 |
| rs34878901 | 19 | 45402477 | 8.90E-01 | 0.004 | 0.029 | 39.8 | 8.70E-01 | -0.007 | 0.042 | 39.3 | 0.000 | 0.024 | 0.83066 | 9.84E-01 |
| rs73936965 | 19 | 45375522 | 9.55E-01 | -0.017 | 0.298 | 0.2 | 9.56E-01 | 0.027 | 0.486 | 0.2 | -0.005 | 0.254 | 0.93954 | 9.85E-01 |
| rs4609015 | 6 | 26407408 | 6.16E-01 | 0.021 | 0.041 | 13.4 | 5.08E-01 | -0.038 | 0.057 | 17.2 | 0.001 | 0.033 | 0.40571 | 9.88E-01 |
| rs10946820 | 6 | 26411188 | 6.15E-01 | 0.021 | 0.041 | 13.3 | 5.08E-01 | -0.038 | 0.057 | 17.2 | 0.000 | 0.033 | 0.40534 | 9.88E-01 |
| rs4712986 | 6 | 26396203 | 6.53E-01 | 0.020 | 0.044 | 12.3 | 5.24E-01 | -0.038 | 0.060 | 15.5 | -0.001 | 0.035 | 0.43559 | 9.89E-01 |
| rs78545860 | 3 | 110857690 | 8.76E-01 | 0.024 | 0.155 | 0.8 | 8.21E-01 | -0.046 | 0.204 | 1.1 | -0.002 | 0.123 | 0.78395 | 9.90E-01 |
| rs10433385 | 3 | 110855748 | 8.74E-01 | 0.025 | 0.155 | 0.8 | 8.21E-01 | -0.046 | 0.204 | 1.1 | -0.001 | 0.124 | 0.78245 | 9.92E-01 |
| rs383483 | 19 | 18171886 | 4.84E-01 | 0.019 | 0.027 | 49.8 | 2.95E-01 | -0.044 | 0.042 | 48.0 | 0.000 | 0.023 | 0.20765 | 9.95E-01 |
| rs7122738 | 11 | 119547189 | 6.71E-01 | -0.068 | 0.161 | 0.8 | 5.55E-01 | 0.130 | 0.221 | 0.9 | 0.001 | 0.130 | 0.46693 | 9.96E-01 |
| rs7250339 | 19 | 45145612 | 9.51E-01 | -0.002 | 0.034 | 24.1 | 9.35E-01 | 0.004 | 0.048 | 26.2 | 0.000 | 0.028 | 0.91859 | 9.97E-01 |

^a^SNP ID (rs#), chromosome location and position are provided for SNP identification

^b^Ordinal p-value for analysis in the Rochester cohort only, after adjusting for gender, assay batch effect, quartiles of age at enrollment, immunization age, time since last immunization to enrollment, and race-specific population stratification eigenvectors

^c^Estimate from the analysis in the Rochester cohort showing the magnitude and direction of the estimated effect on the immune measure; standard error

^d,g^Minor allele frequency refers to the frequency at which the least common allele occurs in the study cohort (%)

^e^Ordinal p-value for analysis in the San Diego cohort only, after adjusting for gender, assay batch effect, quartiles of age at enrollment, immunization age, time since last immunization to enrollment, and race-specific population stratification eigenvectors

^f^Estimate from the analysis in the San Diego cohort showing the magnitude and direction of the estimated effect on the immune measure; standard error

^h^Pooled estimate from the meta analysis showing the magnitude and direction of the estimated effect on the immune measure; standard error

^i^Homogeneity test p-value estimating the homogeneity between the Rochester and the San Diego cohorts

^j^Meta-analysis p-value after adjusting for gender, assay batch effect, quartiles of age at enrollment, immunization age, time since last immunization to enrollment, and race-specific population stratification eigenvectors
